# Of Establishments for the Insane in France, and the Means of Improving Them

**Published:** 1840-01

**Authors:** 


ART. VI.

1. *Des Etablissemens consacrés aux Aliénés en France, et des Moyens de les améliorer. Mémoire présenté au Ministre de l'Intérieur, en Septembre* 1818. Par E. ESQUIROL, &c. &c.—*Paris*, 1838.
*Of Establishments for the Insane in France, and the Means of improving them: a Memoir presented to the Minister of the Interior in September* 1818. By E. ESQUIROL, &c. &c.

2. *Maisons d'Aliénés.* (*Dictionnaire des Sciences Médicales.*) Par E. ESQUIROL, &c. &c.—*Paris*, 1838.
*Lunatic Asylums.* (*Dictionary of Medical Sciences.*) By E. ESQUIROL, &c.

3. *Essai sur les Distributions et le Mode d'Organisation, d'après un Systême Physiologique, d'un Hôpital Aliénés pour quatre à cinq cent Malades; précédé de l'Exposé succinct de la Pratique Médicale des Aliénés de l'Hospice de l'Antiquaille de Lyon, depuis de* 1*er Janvier*, 1821, *jusqu'au* 1*er Janvier*, 1830. Par R. PASQUIER, D.M. &c. &c. —*Lyon*, 1835. 8vo, pp. 52.
*Essay on the Distribution and Organization of a Lunatic Hospital for four or five hundred Patients, according to a Physiological System; preceded by a succinct Account of the Treatment of the Insane in the Hospital of Antiquaille of Lyons, from January* 1, 1821, *to January* 1, 1830. By R. PASQUIER, M.D. &c.

4. *On the Constitution of Asylums, and the Mode of their Management.* By Sir W. C. ELLIS, M.D. (*The Eighth Chapter of his Treatise on Insanity.*)—*London*, 1838.

5. *Total Abolition of Personal Restraint in the Treatment of the Insane. A Lecture on the Management of Lunatic Asylums, and the Treatment of the Insane, delivered at the Mechanics' Institution, Lincoln, on the* 21*st June*, 1838; *with Statistical Tables, illustrative of the complete Practicability of the System advocated in the Lecture.* By ROBERT GARDINER HILL, House-Surgeon of the Lincoln Lunatic Asylum.—*London*, 1839. 8vo, pp. 112.

6. *State of the Lincoln Lunatic Asylum.* (*Fifteenth Report.*)—*Lincoln*, 1839. 8vo, pp. 28.

7. *Sixth Annual Report of the Trustees of the State Lunatic Hospital Worcester* (*Massachusetts*). *December*, 1838.—*Boston*, 1839. 8vo, pp. 88.

8. *Nineteenth Annual Report of the Directors of the Dundee Royal Asylum for Lunatics.*—*Dundee*, 1839. 8vo. pp. 42.

9. *Medical Report to the Managers of the Lunatic Asylum of Aberdeen, for the year ending* 30*th April*, 1839.—*Aberdeen*, 1839. 8vo, pp. 16.

10. *Report of the Directors of the Montrose Lunatic Asylum, Infirmary, and Dispensary, for the year ending* 1*st June*, 1839. 8vo, pp. 22.

11. *State of an Institution near York, called the Retreat, for Persons afflicted with Disorders of the Mind.*—*York*, 1839. 8vo, pp. 19.

12. *Regulations of the Crichton Institution for Lunatics, Dumfries.*—*Dumfries*, 1839.

13. *Statistics of the Bloomingdale Asylum for the Insane.* By James Macdonald, m.d., Physician to the New York Hospital, late Resident Physician of the Asylum. (*New York Journal of Medicine and Surgery. October*, 1839.)

14. *Twenty-ninth Annual Report of the State of the General Lunatic Asylum, near Nottingham, delivered by the Vice-President and Visiting Governors, at their Anniversary Meeting, held in the Shire Hall, at Nottingham, on Tuesday, the 8th day of October*, 1839.

15. *Fiftieth and Fifty-first Report of the Visiting Justices of the County Lunatic Asylum at Hanwell, and Report of the Resident Physician, October* 31, 1839. 8vo, pp. 24-49.

In the construction and organization of a house for the habitation of lunatics, it would seem to be of the first importance to keep in mind that it is intended to be a place of security for human beings, brought by their lamentable malady to such a state that with the force and dexterity of adult age are conjoined the love of mischief and the helplessness of childhood. Humanity steps beyond the consideration of mere security, and adds much that is required for protection, for comfort, and even for enjoyment; and science, deriving salutary aid from these humane arrangements, adds others with the intention of securing relief and promoting recovery.

As every large establishment of such a kind must contain a number of patients whose incoherent or imbecile state, the result or sequence of the form in which the disease first manifested itself, has been found, by long and varied experience, to be little under the control of therapeutic measures; the well-doing of this considerable class of patients depends mainly on the regulation of all circumstances that have a general influence on the health of the body and mind. To breathe good air, to have good food in good quantity, to sleep comfortably, to be clean in person and decently dressed, to take exercise regularly, to be screened from vicious or exasperating excitements, and to be solicited by gentle and frequent efforts to self-control or to mental alacrity, are the points of most importance to such patients, and those to which their superintendent must systematically direct his attention; always remembering that this care is demanded for a class of persons who have the unhappiness to labour under the worst of human calamities. From these circumstances are to be deduced, without difficulty, all the principles that should be observed in the government of lunatic asylums; principles yet of recent acknowledgment, but of stability and truth; the blessed product of that enlightened and universal charity which, although it has not yet flourished equally in every age and clime, has its imperishable root in Christian institutions. The details flowing from these principles may vary infinitely, but the objects must ever be the same. By an observance of these principles a lunatic house may itself become what M. Esquirol has designated it—"an instrument of cure, and, in the hands of a skilful physician, the most powerful therapeutic agent against mental maladies."

But that it should be so implies well-devised arrangements, a careful classification, practical experience, various knowledge, humane dispositions, ample attendance, a superintendence ever watchful, and the continual exercise of a philanthropy which nothing can weary, and a judgment which scarcely anything can disturb. In many of the private and public asylums of this and of other countries, the recommendations of the establishment necessarily fall below this standard; but the difficulty of managing lunatic patients in a private house, or whilst in the bosom of their families, and the want of a general familiarity with the phenomena and treatment of the different forms of insanity, often induce medical men, to whom such deficiencies are best known, to concur, with very little reflection, in sending patients to houses in which they cannot possibly enjoy the benefit of such various remedial means and such enlightened care as their peculiar affliction requires.

Scarcely credible does it now seem that, no longer back than twenty years ago, the condition of lunatic houses throughout France, and, with some exceptions, throughout Europe, should have been such as we find it described in M. Esquirol's historical review of those establishments. Prisons and dungeons were built and dug for them, and chains forged for them. To prevent their dying of sheer hunger was the sum of duty apparently thought due to them; and they were everywhere the victims of ignorance, of prejudice, and of terror. The patients (in France) were found by him covered with rags, lying upon straw, miserably fed, and grossly abused by ignorant and brutal keepers. Air to breathe and water to drink were almost equally excluded from them: they were chained like wild beasts, but in dens into which wild beasts would not have been put. In Germany, as described by Reil, by Joseph Frank, and by Max. Andrée, their condition was equally horrible. In Italy and Savoy the same enormities existed.

It is in vain that we would endeavour to represent even England as forming an exception; for, not to speak of the past mismanagement of Bethlem, the history of one of our provincial asylums, under the superintendence of eminent physicians, and the control of a committee of the first persons in one of the most considerable counties in England, was for the long period of thirty years the scene of every abuse that rapacity and inhumanity could crowd into a single institution. The mere recital at the present day would exceed belief. Suffice it to mention that, among the instances of mismanagement gradually brought to light, were the most aggravated neglect of all medical and moral treatment; every species of cruelty; much gross immorality; every practicable variety of shabby embezzlement and peculation; false reports, in which, especially, the *deaths* were concealed, even so many as one hundred at a time; the occasional disappearance of patients, supposed to have been murdered outright, and returned in the reports as dead, or removed, or cured; and, as a grand and appropriate finale, a very strong suspicion of the building itself being wilfully set on fire, in the hope of destroying some of the books or some of the patients. All these abuses were at length laid bare; but not until many honest enquirers had suffered every kind of insult and obloquy, and death had secured the principal offenders from human disapprobation: for, during the long series of years in which these enormities were practised and suspected, every abuse, every outrage, except the actual murders and burning, was defended by the officers of the institution, and screened from too prying eyes by the most dignified expressions of satisfaction from the committee, who were all the time mystified by false accounts, kept in double sets for the purpose; and to whom, so heedless and confident were they, and to the nominal visiters, even some of the noisome *cells* forming a part of the establishment were actually *unknown*. Now and then, in this long period of blind confidence, a very delicate censure was extorted from them; but it should never be forgotten that for more than twenty years they obstinately and successfully resisted enquiry and reform; although during this time the deaths in the asylum were about 1 in 6.

The institution here alluded to was a kind of epitome of all the evils existing in all the lunatic asylums of the country; but a certain number of the enormities which made up the frightful mass at York were to be found, in various combinations, in almost every place throughout England in which lunatics were confined. M. Esquirol's Atlas of Plates will perpetuate the remembrance of a method of enchaining a poor officer of the navy in Bethlem, at a later period, and even eighteen years after Pinel had struck the chains from off the limbs of the lunatics in the Bicêtre. The poor man, when convalescent, is said to have threatened one of the physicians; and, as ordinary means of restraint were evaded by the patient, an ingenious apparatus of iron was brought from Newgate, and applied to him. This unhappy person is the subject of the last plate; and is represented sitting on a bed of wood and straw, his arms bound, his legs manacled, and a collar round his neck, by which he was fastened, by a chain ten inches long, to an upright iron pillar behind him. The weight of the apparatus was twenty-three pounds; and the patient (it ought rather to be said the prisoner) was unable to walk about or to stand upright, or even to lie down: and in this state he was permitted to remain nine years! Unless this mode of torture was customary at Bethlem, we should presume that this was the history of William Norris, laid before the Parliamentary Committee in 1815. Norris existed twelve years in a state very similar to this, at least; and was at length released by dying of consumption. During much of the time of his confinement he was well enough to read books of all kinds, and could talk quite coherently "on the events of the war." From the information laid before the same committee, we might take other horrible examples; but it would scarcely serve any useful purpose to do so. The only good effect of recalling any of these disgraceful transactions is to prevent too implicit a trust in institutions of the most inviting aspect, when jealously concealed from the public eye.

There is, in truth, scarcely an institution in the kingdom from which the shadow of former evils is altogether departed. We have learned to distrust the fairest outsides. Whole bookfuls of compliments to humanity signalized as unrivalled, and dexterity vaunted as matchless, do not move us one jot. All these things may coexist with great neglect, unseen in the show hours, but pervading every corner of the establishment, and saddening every patient's heart. Scanty diet; want of sufficient air and exercise; windows deeply encroached upon by iron panes, excluding both light and air, neglect of medical treatment in the early stage of the disorder; deficient aid in incidental sickness; early locking up in the evening for twenty-four hours of solitude, and miserable fancies, and burning thirst; Sunday neglect, that keepers and nurses may divert themselves abroad, their number being disproportionately small to the number of patients, and their wages only attractive to the rejected of other occupations; coercion-chairs, in which poor irritable wretches are consigned to brutality of habits and stupidity of mind; frequent, and severe, and long-continued restraint, and not unfrequent blows: these things, thus collected, seem but horrible exaggerations; yet of each of these, singly taken, the lunatic asylum will furnish warm advocates, and few are the asylums in which some, nay, in which many of them, do not exist. Vainly do they boast of the neatness of their halls, and the silence and order of their wards, who have in some unfathomed corner of the house miserable creatures sitting strapped in chairs, with their restless hands in heating and constraining leathern muffs. More vainly still, if, as on too good grounds we fear to be the case, they have others in *chains*, and *kept in chains for years*. "They make a solitude, and call it peace," upon which we pray that the eye of authority may soon be directed. It is a refinement of the unprincipled to avail themselves of the benevolent feelings characterizing the time, for their own unworthy ends; and the spirit of gain, opposed to the Christian spirit by an everlasting incompatibility, may strive, we fear, in these times, to throw over these evils the fair cloak of employment, boasted of as profitable labour; under which cloak, however skilfully its folds are arranged, we shall find that, whilst many are subjected to fatigue, for which both their malady and their diet render them unfit, some are detained long after their convalescence is confirmed; detained because they are useful, and help to support an elaborate delusion. The spirit which can, in these days, prolong violent restraints, is not to be allayed, and therefore never to be trusted. They who have slept sweetly for many an untroubled year out of the hearing of the clanking iron, that all the time has been eating into the soul of some of their poor patients under the same roof, can never be expected to give a hearty concurrence to measures altogether different in their nature. We are too well aware of the impolicy of overstating a cause to peril a cause like that which we are advocating, by exaggerating the faults we denounce. But we have much reason for believing that lunatics are still kept enchained in certain asylums in England; and as to the minor evils, we have still stronger reasons for saying that we suspect these things to exist in many institutions in which, in the place of medical archives, the only carefully preserved books are certain manuscript compositions, inspired by hospitable luncheons and dinners, and of which the authors are enthusiastic but slenderly-informed ladies and gentlemen, who, accustomed to the more elegant condescensions of charity, are at a loss for terms in which to express their admiration of the little they have seen, and the much they have heard, as they hurried through the wards of the poor and the mad.

But so great progress has been made towards a humane and enlightened direction of lunatics, as to leave no doubt that all these lingering evils will be gradually swept away. Five and forty years ago, says Esquirol, lunatics were enchained throughout Europe. Eighty lunatics at the Bicêtre were unchained by Pinel in 1794, and the general treatment was henceforth improved; thongs and scourges were no longer delivered out to the keepers; and the result was, that many lunatics, before deemed incurable, recovered, and that all the rest became quieter and more easily governed. France, he observes, with just pride, was the first nation to offer the spectacle of nearly three thousand lunatics kept in confinement (in and near Paris) without chains, without blows, without unkind treatment: and he quotes, with justifiable disgust, the cold-blooded opinions given by medical authorities in England at a later period, on these points of lunatic discipline. He alludes to numerous inventions, of more or less ingenuity, for confining the arms, legs, and heads of patients, on which we hope it is unnecessary to make any critical observations, the physician appearing likely in our day to supersede the carpenter and the blacksmith in this department of medicine.

A chapter in Pinel's admirable work is on the custom of flogging lunatics, as a means of cure: *Sur l'usage de frapper les Aliénés, comme un moyen de les guérir.* Every precept, before his time, was enforced by a rap on the knuckles, a blow on the head, or a lash on the back, given with a severe and virtuous gravity. There is astonishing convenience in this kind of despotism; and mankind always zealously cling to it. For all but its victims, its recommendations are overpowering. Eternal honour to Pinel! who, first of all in Europe, raised his voice against these atrocities, and pointed out the excitements they produced, and contrasted them with the calm that ensued on kind and compassionate treatment. Yet it moves our surprise that, after all his efforts, these evils should still have maintained their ground for so many years.

When M. Esquirol made his tour of inspection, lunatics were still commonly confined in hospitals for the reception of old and imbecile persons, with patients affected with the itch or with syphilis, with children, prostitutes, and criminals. This was the case in no fewer than thirty-three of the principal towns in France. In some other towns they were placed in the depôts of mendicity; and in others in the prisons, where, less favoured than the prisoners, they were not enabled to mitigate their condition by the trifling product of their labour, work being denied to them. Wherever placed, their sleeping-rooms, or rather their cells, were damp and unwholesome, and their food the refuse of the coarsest victuals given to criminals or paupers; whilst, in point of bed-comfort, the dogs of the establishment were better lodged than they. In almost every place of this kind the unfortunate beings were chained: it saved servants, it rendered better management superfluous—it even prevented the expense of strait-waistcoats. Sometimes a chain was attached, in large sleeping apartments, to every bed; and under their clanking the unhappy patients nightly sought repose. Against all the particulars of this shocking system, the medical men, much to their honour, made repeated protestations; but the authorities were always afraid of the insane, thought insanity incurable, and conceived that, if actual starvation was avoided, all was done which the miserable wretches required.

Whatever pleasure we should have in fancying such evils no longer to exist in any European country, we cannot forget that in Genoa, even at this time, about 250 patients are described by Dr. Lee as being confined to ill-ventilated wards, "which they are never allowed to leave until they die or are dismissed the hospital." In the women's ward many are chained by the hands and feet; and the mortality of the institution is enormous.

The reform of such establishments has begun, and made considerable progress; but the great objects of philanthropy are seldom rapidly effected; the hostility of the unfeeling and of the prejudiced is never overcome by one assault; and unless the courage of the benevolent be accompanied by equal patience and vigilance, the work still remains long incomplete.

M. Esquirol has urged with great earnestness the institution of separate hospitals for the insane; a subject at present occupying the attention of the French government. In doing so, he has pointed out with much force the importance of having a few large establishments, instead of a small one for each department; among several reasons for which he represents the difficulty of finding a great number of physicians fitted by their acquirements and disposition to superintend them.

" It requires," he says, " a mind of particular cast to cultivate this branch of the art of healing with advantage: the physician must have much time at his disposal, and make as it were an abnegation of self. It is not likely that a physician, already enjoying a great reputation and a large practice, will take charge of a small hospital, that will occupy all his time, expose him to dangers, and offer him but few chances of success. In fact, he who would be useful to lunatics should visit them several times in the day, and even during the night; he should not content himself with a visit made in the morning, as is the custom in common hospitals. But in a small hospital, what hopes of cure will sustain his courage? Charged with the care of an hospital containing from thirty to sixty lunatics, scarcely ten will offer any prospect of recovery; and of these ten, under favorable circumstances, he may cure five. And yet this small number of cures should not be considered discouraging, when it is remembered that in the departmental asylums the mass will consist of incurables, and the physician can only calculate on few favorable cases."

In this report, M. Esquirol gives a description of the plan for a lunatic asylum, which appeared to him, after ten years' consideration, the most convenient; and in conformity to which, we believe, several of these buildings have subsequently been erected. The general plan is that of a central building for the officers of the establishment, with wings for the male and female patients, so arranged as to admit of divisions and subdivisions of the patients; the separate parts forming quadrangles with a central square encircled by a gallery. The furious, the tranquil, the melancholic, the convalescent, are, by this plan, separated from all the other patients. This is very nearly the plan of the Bicêtre, and of the asylums of Rouen, Montpellier, le Mans, Nantes, and Marseilles.

Some arrangements, scarcely existing at the time, but which will now be acknowledged to be obviously necessary, are suggested in the report, such as that the compartments of the building destined for the furious should be of a more solid construction than the rest; that the floors of the rooms of patients who cannot be induced to cleanliness or decency in their habits should be of stone, and sloping towards or from the door; and that the convalescent department should be like a private house. In these and all other respects great diversity of plan obtains in the different English asylums. The strong doors are lined with iron in some, and in others merely consist of wood; whilst in some the doors are only of ordinary strength. The convalescent department is seldom complete: it is indeed difficult to render it so, particularly in small establishments, where the number convalescent at any given time is very inconsiderable. Many very forcible reasons are given by M. Esquirol for placing all the patients on the ground-floor. The inconvenience and dangers of stairs, and the difficulty of having up-stairs rooms properly cleaned and attended to, are among the disadvantages of buildings of many stories; and the greater liberty of the patients, and greater facility of going out of doors when they please, or when free exercise in the open air is desirable for the alleviation of a paroxysm, as well as the readier attentions and inspection thus promoted, are some among the many advantages of the patients being thus placed. The fatal objection to this plan, implying so many separate buildings of one story, a kind of village in fact, is the great expense attending it. It is, however, as M. Esquirol well observes, deserving of consideration, whether economy is not best consulted by arrangements which most facilitate the cure of the inmates; a mode of reasoning too uncommon in relation to this subject.

The report concludes with a recommendation that to each asylum there should be an administrative council, consisting of the prefects of the departments associated in establishing it, of subscribers, the procureur-general, the principal gentry, &c.; the director and physician of the asylum having, as members of the council, only "a consultative voice." The director and the physician to be named by the minister, on the presentation of the council. A steward, a chaplain, an apothecary, and a superintendent to be appointed by the council, on the presentation of the committee of administration. This central committee to be formed by the minister of the interior; and with it the directors and physicians of all the asylums to correspond. The committee to make an annual general report.

The memoir entitled *Maisons d'Aliénés* is an enlarged and amended edition of the article bearing the same title in the *Dictionnaire des Sciences Médicales.* Of this memoir the introductory portion is entirely historical, and we shall pass it over, curious as it is, without comment. We are glad, however, to quote an opinion of much interest to superintendents of lunatics, coming from so great an authority as M. Esquirol, and which occurs incidentally when he is reciting the instructions given by Colombier for the reform of the Hôtel-Dieu, under Louis XVI., in 1785. It is, we think, a commonly received notion in England, and probably in other countries, that those who have the charge of the insane are prone, in consequence of that association, to become maniacal or imbecile. This opinion is expressed in Colombier's instructions, and the Bicêtre and the Salpêtrière are referred to for examples. On the subject of this alarming belief M. Esquirol says, that in the course of forty years' experience, he has seen nothing which confirms it. We presume the truth to be, that the constant superintendence of lunatics is an occupation somewhat harassing and exciting; and that if the asylum is large, and its duties are conscientiously performed, and the superintendents are excitable, the nervous system may be somewhat severely impressed by continual anxiety, intermingled with the frequent and unavoidable agitations of a house in which so many persons are, by the nature of their malady, exposed to so many accidents. Nearly all the superintendents and attendants of whom we have made enquiries relative to this point describe themselves as having been at first overwhelmed by the duties of their office; and, even after long habituation to them, as being now and then so far disturbed or subdued as to render a short absence, although only the absence of one day, indispensable to the recovery of their composure, or of their cheerfulness. There may be reason to fear that in very impressible constitutions, such effects, by frequent repetition, would become more serious. Much, however, depends upon the temperament; and those who undertake to cure unsound minds should possess some skill in managing their own.

In the brief, but distinct and interesting, notices given of the state and history of the principal lunatic asylums of France, we read almost a uniform record of past neglect and recent and happy reforms. On the progress of the latter M. Esquirol is entitled to look back with some self-complacency; his writings, his personal visits, his reports, his plans, his suggestions to the government, to committees, to architects, and to physicians, having materially contributed to many desirable ameliorations. The fuller details, the results of all his observations during his long and useful career, are promised to the public in a separate work, which he is about to publish, on lunatic establishments. It is evident that the uniformity of the amendments in the French institutions has been facilitated by the circumstance, that very few lunatics in France are confined in private asylums. For instance, in the department of the Seine, although there are twenty private asylums, the whole number of patients distributed among them does not exceed 400; whilst the three public asylums of Paris contain nearly 3000. In England and Wales, M. Esquirol was informed by Sir Andrew Halliday, in 1832, that the number of patients in the public asylums was 4077, and that there were not fewer than 2453 in private establishments. M. Esquirol laments that there are yet in France so few institutions entirely devoted to lunatics; and that there is nowhere any special and clinical hospital for the instruction of young physicians in their treatment. "Will our country," he says, "set the example?" We may echo his question in this country; where, as we have already had occasion to remark, (Number XIII. p. 34,) no direction of any public medical school has yet been found enlightened or courageous enough to add the opportunity for the clinical study of mental disorders to the other advantages of medical education. There are, we are well aware, some difficulties in the way of such arrangements; but we cannot think them insuperable; and the good to be obtained is very great, and very important to the public.

The want of buildings so separately constructed and arranged as to admit of a proper division of lunatics, according to the character and stage of their malady, is observed by M. Esquirol to be a very general defect, even of institutions entirely devoted to the insane. This fault is, he says, very remarkable in the most boasted establishments of England, and in many parts of Germany and of America. It doubtless arises from a too eager economy. There are very few houses in which the furious are rigorously isolated from the tranquil; the epileptic patients and those affected with accidental disease are not always separated from the rest; nor are the convalescent removed from those who are yet under treatment for acute forms of mental disorder. In many houses, particularly in England, he remarks, the rate of payment nominally determines the patient's accommodations; but it can only really do so for those who are cleanly and quiet: and he does not approve of the buildings for the poorer patients being placed so as to show them the superior comforts of the richer. He thinks that the only basis of the classification should be the character and stage of the malady; but it surprises us that he should not take into consideration the effect produced on the minds of the patients by the presence or absence of comforts to which they have been accustomed. To circumstances of this kind few lunatics are quite insensible; and, after recovery, their reflections may be materially affected by them.

M. Esquirol disapproves of lunatic asylums being placed in towns, for obvious reasons; and he lays great stress on the necessity of having a sufficient number of courts, and of sufficient size, and of a cheerful aspect, for the exercise of the different classes of patients. He reprehends the practice of arranging the patients in lofty buildings, some in apartments half under ground, and some in rooms higher up, from which they cannot freely and readily descend. He prefers that the apartments should be on a level with the ground, not only for reasons which have already been mentioned, but because this plan gives them so much more freedom, without the annoying accompaniment of unlocking and unbolting doors whenever the patient is to walk out. His observations on the too common fault of constructing cells solely with a view to furious lunatics, who scarcely constitute ten out of a hundred cases, are such as are not always remembered, we think, at present; and he points out the propriety of noiseless locks, flat bolts, and windows which admit air and the cheerful light of day. It would be most ungrateful to forget the philanthropic and enlarged principles promulgated by Pinel at the close of the last century, or the sensible and humane observations on this matter, published by Mr. Samuel Tuke, in his Description of the Retreat near York, five-and-twenty years ago; works in which many of the most important principles of the treatment of lunatics are enforced in a manner so eloquent and so unpretending as to be worthy of all imitation. "Many errors," observes Mr. Tuke, "have arisen both in the construction and management of asylums, from an excessive attention to safety; and it has been made an excuse for much improper treatment, and for much vicious neglect on the part of the attendants." He mentions a visit he made to a house for insane persons, in which security was made a *primary* object; and where he found three of the keepers, in the middle of the day, earnestly employed in playing cards. We think, indeed, that a pretty correct estimate of the general management of a lunatic institution may be made by observing the number actually under corporeal restraint. It is here as in a regiment; where severe punishments are frequent, the commanding officer will generally be found inefficient.

In this particular there is apparently no asylum in England which presents so remarkable a model as that of Lincoln. Of all the works that have appeared on the subject of lunatic houses since the publication of Mr. Tuke's account of the Retreat, there is none which contains matter more deserving of attention than that recently published by Mr. Hill. His lecture is little more than a simple commentary on the resolutions of the board of the management of the Lincoln asylum for twenty years past, during which period, under the superintendence of Dr. Charlesworth, and, latterly, with the vigilant co-operation of Mr. Hill himself, as house-surgeon, almost every kind of bodily restraint is stated to have gradually fallen into disuse as superfluous, or worse than superfluous, a mere substitute for want of watchful care. This great alteration of treatment, opposed to long practice and strong prejudice, and even to opinions yet very generally entertained, has only been effected, it is said, by the most careful adoption of all the parts of a system of constant superintendence, of well-preserved classification, and of humane and effective practical management. Very happily for the inmates of the Lincoln asylum, the managing board seems ever to have acted cordially with the medical officers; regulating even the building and arrangement with a view to curative effects rather than to mere short-sighted economy. Thus, besides other advantages, a classification has become practicable which answers the purposes of comfort, encouragement, safety, and cure. A well devised night-watch suffices to prevent the occurrence of suicides and of various irregularities, without the necessity of irksome and irritating night-restraints. The attendants are numerous, selected for good temper, and of sufficient bodily power; so that every patient is watched, and knows that he can be controlled; and yet every patient enjoys a great degree of liberty, and none are tortured by continual coercion. By means of sash-doors, the attendants themselves are liable to continual inspection, and little is left to their undisciplined temper or caprice. No corporeal restraint is inflicted without an order from the medical attendant; ill-usage of a patient is immediately punished; and a room is kept in which all the instruments of restraint are open to examination, so as at once to show their nature and the number in actual use at any time; whilst each application of them is regularly entered and reported. We know no more interesting document than the following, which is a summary of the tables appended to Mr. Hill's Lectures, showing the several instances of restraint, coercion, and seclusion, used in the Lincoln Lunatic Asylum, from March 16th, 1829, to March 22d, 1837, "*when the practice was wholly discontinued.*"

"SUMMARY OF THE PRECEDING TABLES.

| Year. | Total Number of Patients in the House. | Total Number of Patients restrained. | Total Number of Instances of Restraint. | Total Number of Hours passed under Restraint. |
|---|---|---|---|---|
| 1829* | 72 | 39 | 1,727 | 20,424 |
| 1830 | 92 | 54 | 2,364 | 27,113¾ |
| 1831 | 70 | 40 | 1,004 | 10,830 |
| 1832 | 81 | 55 | 1,401 | 15,671½ |
| 1833 | 87 | 44 | 1,109 | 12,003½ |
| 1834 | 109 | 45 | 647 | 6,597 |
| 1835 | 108 | 28 | 323 | 2,874 |
| 1836 | 115 | 12 | 39 | 334 |
| 1837 | 130 | 2 | 3 | 28 |

"After deducting the number of patients introduced in the above Table more than once in the years 1829-30-31-32-33-34-35, and also the readmitted cases

* From March 16th.

within the same period, the actual number of patients restrained in the course of such seven years was 169.

"Of these 169 there remained in the house, at the end of such seven years, 43.

| | |
|---|---|
| Of these remaining 43, there were discharged from the books, during the years 1836-7, *not having been restrained at all* during any part of such two years . . . . . . . . . . . . . . | 11 |
| "...................... having been restrained only for about *seven hours* during any part of such two years . . . . . . . . . | 2 |
| "...................... remained in the house December 31, 1837, *not having been restrained at all* during any part of such two years . | 29 |
| "...................... having been restrained *once only* (for about nine hours) during any part of such two years . . . . . . . | 1 |
| | 43" |

We are perfectly aware that Mr. Hill's statements will be received in many lunatic asylums with surprise, and even with incredulity. The proportion of sane keepers to the patients is so very small in most of those institutions, and so much habitual assistance is calculated upon from imperfectly recovered minds, that the effects to be expected from a better system of watchfulness has scarcely yet suggested itself to the imaginations of the directors and superintendents. Perhaps, indeed, it is difficult for any one not familiar with the working of such a watchful system to believe it sufficient in *every* case, without the old physical restraints. If the Lincoln asylum can present a model of this kind, which all may visit and examine, the services of Dr. Charlesworth to the cause of humanity and in behalf of the insane, already considerable, will only be second to that of him who first released them from their chains. We know that many superintendents, not open in any degree to the charge of inhumanity, are of opinion that the occasional infliction of corporeal restraint, for a very short period (for a few hours for instance), is followed by such salutary effects as entirely to take away from it any objection on the ground of hurtfulness. Thus applied, they say, it abbreviates the paroxysms of many mischievous patients, and becomes a powerful persuasive to self-control. They suspect, also, that if such means are wholly laid aside, the keepers, however vigilantly watched, will take even more objectionable methods of subduing the patients, and produce a deceptive appearance of tranquillity by means of fear, excluding the patients not only from all violent manifestations of their malady, but almost from all manifestation of sensibility and of mind. The tranquillity of the Lincoln asylum is certainly one of its most striking peculiarities. There is scarcely a noisy patient to be found in it. The refractory ward is scarcely refractory; and the patients move about less, and talk less to visitors, than in any other asylum which is known to us. If patients, therefore, are as freely and indiscriminately admitted into that institution as into others, and if they are not *awed* into their submissive and almost torpid aspect, the effect of mere watching, and of the consciousness of being watched, is powerful indeed. The experiment of abolishing all restraint is too important a one to be rejected or received without free question and enquiry; and this appears to be encouraged, and even courted, in the Lincoln asylum. At all events, the evils of excessive restraint are always found to be numerous and shocking, and the object of every physician ought to be to abolish it to the utmost extent consistent with the security and the amendment of the patients.

The fifty-first report of the County of Middlesex Lunatic Asylum, at Hanwell, shows that the managers of that large institution, containing 800 patients, have been the first to follow the example set by Lincoln. A table is appended to the report, containing a list of those in restraint each day for the four months of July, August, September, and October. The greatest number at one time, eighteen, was in July; it is seen to have been gradually reduced, until, in the middle of August, we find none in restraint. A few exceptions occur later in the list; but not one after the 20th of September. This has only been effected by making a sufficient increase of the number of attendants to ensure a very vigilant superintendence of the patients; and it is thought the general good effects of the change are already visible in the asylum.

In too many cases, we suspect, the mode of proceeding with lunatics in the early period of their affliction is the cause and not the consequence of the most alarming or the most offensive characters of the malady, which characters become exaggerated in its more advanced stages. Fits of violence are met by violent restraint, and an aggravation of the violence follows. Close bodily restraint induces uncleanliness; and the helpless creatures, disabled from any effort to maintain decent habits, are still more closely restricted, on account of the unhappy effects which the first restriction occasioned. Not a day passes, where this system prevails, in which some passionate keeper or some impatient nurse does not strap up some unhappy patient closely, and hastily, and fiercely; and the period of duration of such restraint, unless carefully and daily reported, (and where is this done?) is very uncertain, extending often to twenty-four hours, and galling the poor victim day and night.

Well worthy would it be of the managing committee of some great asylum to institute a normal school for keepers and nurses, where they might be rigidly accustomed to rely on the restraining power to be exercised by the proper application of a reasonable mind over minds of every shade of infirmity and disorder. Full, careful, patient, systematic experiment has not yet authorized any one, by its failure, to pronounce moral restraint insufficient for the control of any lunatic asylum in the world. Until such experiment is made and long persevered in, we fear that occasional bodily restraint must be resorted to. At the same time, we believe such restraint, if ever salutary and remedial, when most deliberately imposed, for the shortest period, and removed with the most considerate manifestation of a wish to give relief, which still we strongly doubt, will always be found to be inefficient and hurtful, in proportion to its long continuance; and especially hurtful if habitually resorted to for punishment rather than for protection. Of its real benefit at any time, for however short a period, and in any circumstances whatever, we are extremely sceptical. We say its benefit, not its convenience, which is often mistaken for benefit. Mr. Hill goes further: but those who object too hastily to his opinions should consider the qualifications with which he accompanies them: "*that in a properly constructed building, with a sufficient number of suitable attendants,* restraint is never necessary, never justifiable, and always injurious, in all cases of lunacy whatever." (p. 21.) If any view of restraint could palliate its disadvantages, it would be that in which it is set in the Dundee Report; as a measure to which there is no alternative but terrifying and paralyzing the mind. But we do not believe that such is the alternative; and although the Dundee Report will be and is appealed to by all the admirers of the old system, from the strait waistcoat of the present day to the iron collar of Bethlem, and the chains of the Bicêtre, we are glad to find that there is actually no restraint at all imposed on the inmates of the Dundee asylum, "with the exception of instances of very violent excitement;" which instances, we learn from the same report, are as many as four or five, on an average, in 150 patients, a number which seems small, but is relatively considerable. If four or five require restraint at Dundee out of 150 patients, there should be always from twenty-five to thirty in restraint at Hanwell, which, in the present circumstances of that institution, would be looked upon as an enormous and even disgraceful number. Of the 800 patients there, the most bigoted admirer of sleeves and leg-locks would not be able to find a decent excuse for restraining more than three or four at any one time; and the actual fact is, that in that large institution, since the middle of August, there have never been two patients in restraint at any one time; and that out of 470 women there, not one has been in close restraint during the whole of that period. Nor do we learn that any unusual outrages or serious accidents have been the result of this emancipation. Some bedding, we understand, has been destroyed; some windows have been broken; a blow has now and then been given; and an attempt at escape has occasionally been made; but so far from these irregularities being on the increase, they are thought to be gradually diminishing, with the mortifying and tormenting restraints which it is more than half suspected was the cause of them. It is even thought that a blander character and manners begin to pervade the wards. Time will show how true this is, or how delusive; and those who incur the risk of making this great experiment will not, we venture to say, shrink from abiding the result.

Without endeavouring to proceed to the extent of the abolition of corporeal restraints, the character for humanity obtained in lunatic asylums appears to us to be little better than a kind of quackery. The quiet patients are exhibited in triumph; sagacious head-shakings are made over those sitting in the chairs of restraint, like so many beasts in an unclean stable; and the worst patients are not always shown. Besides which, it is the custom in a few asylums, a custom gravely sanctioned by the late Sir William Ellis, who was, we believe, the originator of it, to confine every unfortunate epileptic patient in bed at night, by a strap round one wrist, fastened to the side of the bed, to prevent the patient turning on his face in a fit. Thus ten patients, for the most part of a peaceable and inoffensive character, out of every hundred, are confined by straps for twelve hours out of twenty-four every day of their lives. We believe this practice to have been founded on a very limited acquaintance with the phenomena of epilepsy. But if we were to admit the fact of the turning of epileptics in a fit,—which we are far from admitting,—we should doubt the propriety of the general restraint of epileptics based upon it. "The severity of an asylum," well observes Dr. Charlesworth, "does not, as is supposed, consist in the outrage, blows, and active ill usage occasionally brought to light, and which may be prevented by a superintendent having any claim to humanity or attention, and who has eyes to see bruises and a voice to ask their origin. Its torturing effect lies in the aching of limbs forcibly detained in one position, especially during the night, forbidden the ease and alleviation of change, with confined irritability for which nature has opened the vent of free motion, monotony, the feeling of oppression, surrounding miasma, contempt, and neglect:—all much more keenly felt than occasional violence, and sometimes prompting fatal acts of revenge or despondency." (*Lincoln Report*, p. 5.)

It is a most serious addition of the occasional misery of an epileptic, never to have a night of ease: for either the patient cannot turn comfortably about when confined by the strap, or the strap is useless. If the patient is liable to fall out of bed, the strap is objectionable, as the whole weight would be dragging at one arm. Lowering the beds of epileptics, as at Bethlem, would be better.

Those who speak in terms of eulogy of the moral advantage of even temporary restraints cannot divest themselves, one would think, of the apprehension that restraint once resorted to, and found convenient, will be not temporary, but continued for a hurtful period of time. To walk through a ward in which there is one noisy patient, and to order the patient to be instantly put in restraint, may pass for excellent discipline in the eyes of a hasty, frightened visitor; but if the visitor remained to see the order put in force, what would he behold? First, an ineffectual attempt of one or two keepers to effect the restraint; then a greater power brought to bear on the victim; and a scene of struggling, striking, kicking, biting, spitting, swearing, and screaming, which frights the whole ward from its propriety. Let him go again in an hour, and he will find the patient still noisy, shouting, and cursing all the powers that rule the asylum. In three or four hours it is still the same. At length, perhaps, the patient becomes silent. Happy it will be if even then restraint is removed and food is taken to him, or water to allay his thirst. But suppose no restraint is put on at all? Let the noisy patient's attention be diverted by being taken into the airing court; or, at the worst, let him be shut up in his room, the windows well secured, and the bedding removed. In that case we venture to say that in a much shorter time tranquillity will be restored. There will have been no struggle; and the punishment will leave no rankling sense of mortification; and the other patients will not be excited. Still, even in this case, the seclusion should not be prolonged. Many times, however, without any seclusion at all, a sensible keeper may so manage a refractory patient, that if the visitor were to return to the ward in a quarter of an hour, he would find the man whom he would have consigned to restraint quite tranquil, and civil, and cheerful.

In the acute stage of mania, one patient will doubtless require all the attention of one keeper, and perhaps of two, and for several days, or weeks, if the patient is not shut up. But in a well-regulated asylum this occasional attendance on troublesome patients should be a part of each keeper's duty, and taken by each keeper in turn for one or two hours. The attendants, if it is pretended that the patients are to be *cured*, should be sufficient to spare a keeper for an hour or two from any ward for such occasional duty. Then it will be seen that the acute stage (no other means of control being omitted) is not of such long continuance; and that the patient's temper is unspoiled. The patient will gradually subside into tranquillity, and retain a grateful sense of what has been done; every trifling incident in their management being commonly remembered by them. At the same time, the other patients will not have been familiarized with the wretched spectacle of a poor creature dancing about in a strait waistcoat, like an intoxicated mummy; nor will their ears have been assailed with shrieks and curses from the maniac's cell. They will even, to some extent, appreciate the kindness and patience shown to the new comer; for they are keen observers, and have not forgotten their own sufferings.

In almost every asylum there are patients who will destroy their clothes, and some who cannot, it is represented, be persuaded to wear them. In the latter case we are glad to see M. Esquirol speak in disapprobation of forcibly confining the patient. One of the most unsatisfactory customs in some lunatic establishments is to exhibit these unfortunate creatures strapped down in chairs, and to comment, in moving terms, on their violence. The remedy, M. Esquirol says, is as bad as the evil. A dress of strong materials, with a strap round the waist, the dress being entirely fastened behind by a small lock (as done at Hanwell—see *Report*) can scarcely be removed by the patient. Boots and shoes may be easily fastened on by a similar contrivance, and in other cases it would be better to let them for a time be without their clothes, secluded of course from the gaze of visitors, than to add to their irritation, when possibly the wearing of clothes is a source of torment which they can only manifest by tearing them off. Chaining a poor frightened lunatic to his bed, also, when he perhaps thinks the bed full of snakes and scorpions, is ill-judged and cruel. If he persists in sleeping on the floor, having such notions, it is better to let him do so, and after a time he will be reconciled to his bed. It will doubtless be found that there are patients so ingeniously destructive as, with the help of hands and teeth, to tear up every article of clothing that can be devised. The absolute prevention of this can only be effected by putting the patient on straw, with body, and arms, and legs strictly and painfully confined. But are a few blankets, or a gown and cap or two, to be deemed of more value than the immunity of a poor creature from torture, of which the effect is only to subdue, and not to cure?

Of all the sights that can afflict a humane spectator in a lunatic asylum, none is so wretched as that of the patients who are confined in the wooden boxes, or coercion-chairs (a combination of a close-stool and the ancient watch-box), where they sit from morning to night—nay, we fear, from month to month. So revolting is this custom, that it is difficult to persuade one's self it can ever be indispensable. The experience of the Lincoln Asylum, and, more recently, that of Hanwell, with its 800 pauper lunatics, encourages a hope that there are few cases, or none, in which the uncleanly may not be brought to decent habits, and the epileptic guarded, and the furious controlled, by better methods. But to effect these ends, which no superintendent should rest satisfied until he has tried to effect, the keepers must be patient, and very watchful, and sufficiently numerous. We sincerely trust it may be found practicable to abolish from all asylums an invention which we are loath to believe a needful accessory. It is no part of our wish to reflect in such a manner on any parts of the practice in asylums as to give offence to those whose days are devoted to the severe duties of such places; but it behoves them well to consider what might be done by a more liberal system, as regarded the keepers under them, and to appeal to the governing bodies for assistance towards improvements, which every man of humanity must wish for. There is no passage in the appendix to Mr. Hill's Lecture which we have read with more pleasure than the following: "Ordered, That the chairs used formerly for the double purpose of night-chairs and restraint (long fallen into disuse) be worked up." This working-up, and the formal destruction of iron hobbles and handcuffs, and even of strait-waistcoats, which we notice from time to time in the minutes, constitute triumphs in which we earnestly hope it may be found possible for all superintendents to share.

One of the items of recent expense, and which became a subject of comment and censure in a large asylum in the neighbourhood of London, was, "For six deal restraint chairs, £30." The asylum previously possessed thirty-five. At the present moment there is not one in use in the whole of the institution. Two hundred pounds worth of restraint chairs is thus thrown away. But the poor creatures who sate all day in those disgusting chairs may, it is said, be seen jumping about the wards like liberated children; not yet sure, when approached, that a blow is not coming, and yet shrinking with such expectation, and deprecating the expected cruelty in simple, but in touching words; but gradually acquiring confidence, and regaining the almost lost traces of humanity.

They who endeavour to abolish restraints must be warned that they will find much opposition on the part of keepers and nurses; that formidable bills for panes of glass will for a time be displayed before them; and blankets and counterpanes will be daily torn to shreds and scattered in the air. Periods of more difficulty and of temporary alarm and distrust must be expected, in which the superintendent will dread, most of all, the being driven back to use the old restraints; until experience and patience have devised and strengthened better means of controlling violence and preventing irregularity. To ensure the successful abolition of a system, the cruelty of which is, without all doubt or question, condemnatory of it, there must be substituted, for physical restraint, mental control, vigilant watching, care to repress violence, to allay irritation, and to heal quarrels. The attendants must be well versed in the readiest mode of overcoming violent patients; not struggling singly with them, a practice always threatening injury to at least one of the parties, but putting an end to opposition by combination; dexterously depriving arms and legs of inconvenient freedom of action; carefully removing missiles that may be rendered available to the patient's fury; and promptly and calmly bringing every fertility of device to meet every variety of difficulty. If patients are violent, they may be secluded for a short time; if they break windows, the shutters may be secured; if they tear bed-clothes, the bed-clothes may be covered with or sewed up in strong untearable materials; if they pull off or destroy their dress, strong dresses may be given, which will tire out the tearing propensity, secured round the waist with a belt of leather, having a small lock; if they strike, their hands may be enveloped by soft boxing gloves. Their superfluous activity must be exhausted by exercise out of doors. In short, anything should be tried rather than confinement of limbs, the sure parent of uncleanliness never to be cured, and ferocity never to be tamed. And still, for years, the system may not work completely; for many of the subjects of it will be those who have been already vexed with every kind of restraint; have danced for days in strait-waistcoats; have been strapped and howling in bed for weeks together; or have sate in coercion chairs for months. No system can restore some of these victims. They must live out their lives in misery, whereof no small part has been of man's creation.

Much trouble must be taken, some sacrifices must be made, great anxiety must be endured, before the end can be achieved. Among other surrenders to be made by vanity, the glory of silent wards must be given up; for patients, who are happy, are not uniformly quiet. To fill a basement story with the inconvenient patients, to turn out the noisy into dismal courts, and to show an empty gallery; these are not parts of the system now advocated. Good conduct being uniformly enforced, the patients must wear the air of freedom as well as possess it. They will not sit wholly passive and silent, as so many beings with whom the world has no further concern; but will approach cheerfully or with vivacity, and talk to the visitor with confidence. The spruceness of the showhouse may be wanting; but the proportion of patients progressively advancing to health of mind, will be greater than it was under the ancient repressions.

As respects the actual attainment of these results, we cannot but think that the circumstances stated in the Hanwell Report afford a reasonable prospect of it. The progress already made is described in a few paragraphs, which, proceeding, after evident deliberation, from an asylum containing eight hundred pauper lunatics, almost wholly of the poorer classes, have certainly some claim to attention.

"The article of treatment in which the resident physician has thought it expedient to depart the most widely from the previous practice of the asylum has been that which relates to the personal *coercion* or forcible restraint of the refractory patients. Without any intention of derogating from the high character acquired by the asylum, it appeared to him that the advantage resulting from the degree of restraint permitted and customary in it, at the period of his appointment, was in no respect proportionable to the frequency of its application, that the objections to the restraint actually employed were very serious, and that it was in fact creative of many of the outrages and disorders, to repress which its application was commonly deemed indispensable, and, consequently, directly opposed to the chief design of all treatment, the cure of the disease. The example of the Lincoln asylum, in which no patient has been put in restraint for nearly three years, came also powerfully in aid of attempt to govern the asylum at Hanwell by mental restraint rather than by physical.

"Such an attempt could not be extended to all cases without some immediate inconveniences. Attendants accustomed to rely on the easy help of close restraints, were reluctant to abandon them; and unexercised in the resources without which their abolition produced inconveniences, which they were not likely nor able to compare with the remote evils produced by their continuance. Nor would the resident physician yet presume to say that strong restraint may never be required, but he begs to lay before the visiting magistrates a simple statement of the progress of an attempt to do without it. By a list of restraints appended to this report, it will be seen that the daily number in restraint was in July so reduced that there were sometimes only four, and never more than fourteen, in restraint at one time; but that since the middle of August there has not been one patient in restraint on the female side of the house, and since the 21st of September, not one on either side.

"There have, however, been occasional and brief instances of restraint, unsanctioned in some cases by the physician, and which do not appear in this table; but it correctly represents the total absence of continued restraint during the whole period since August 9th. For patients who take off or destroy their clothes, strong dresses are provided, secured round the waist by a leathern belt, fastened by a small lock. For some who destroy the collar and cuffs of their dresses with their teeth, a leathern binding to those parts of the dress is found convenient. Varied contrivances are adopted, with variable results, for keeping clothing on those who would otherwise expose themselves to cold at night; and warm boots fastened round the ancles by a small lock, instead of a button or buckle, are sometimes a means of protecting the feet of those who will not lie down. As it is now and then necessary to confine the hands when a blister is applied, to prevent its removal; and as this, like all other temporary restraints applied with the justifiable plea of protection," is generally abused by being too much prolonged, or unnecessarily severe, a kind of cape, as a covering for a blister, capable of being secured over it, has been thought of, and will no doubt be found practicable. Those who are in the habit of striking suddenly, tearing the bed-clothes, &c., sometimes wear a dress of which the sleeves terminate in a stuffed glove, without divisions for the thumb and fingers. But no form of strait-waistcoat, no hand-straps, no leg-locks, nor any contrivance confining the trunk or limbs, or any of the muscles, is now in use. The coercion-chairs, about forty in number, have been altogether removed from the wards; no chair of this kind has been used for the purpose of restraints since the middle of August.

"It may be considered yet too early to pronounce a positive opinion on the general effects of these measures. In so large an asylum, filled with pauper lunatics, the means of mere mental control must always be limited, and the discontinuance of cruel restraints may only slowly be appreciated by the patients. But the resident physician is inclined to believe, after as careful observation at all hours as the space of a few months has permitted, and notwithstanding some peculiar difficulties, that the noise and disorder prevalent in some of the wards has already undergone diminution; that instances of frantic behaviour and ferocity are becoming less frequent; that the paroxysms of mania to which many of the patients are subject, are passed over with less outrage and difficulty, and that if cases are yet seen, which appear for a length of time to baffle all tranquillizing treatment, they chiefly, if not exclusively, occur in acute cases, the symptoms of which would be exasperated by severe coercion, or among those who, having been insane many years, have been repeatedly subjected to every variety of violent restraint.

"With respect to the discontinuance of the restraint-chairs, he may speak more confidently. Several patients formerly consigned to them, silent and stupid, and sinking into fatuity, may now be seen cheerfully moving about the wards or airing-courts; and there can be no question that they have been happily set free from a thraldom of which one constant and lamentable consequence was the acquisition of uncleanly habits."

The habitual comfort and cheerful alacrity of the attendants, who live in the atmosphere of excitement and anxiety, of which we have spoken, must by no means be overlooked. It claims even particular attention. Upon them it mainly depends, and ever must depend, whether or not *habits* of order, decency, and good behaviour grow up and strengthen. Unless *they* are well trained, the mental plan, however enlarged, cannot supersede the physical plan: and the new discipline must be established, even for a few years, before its effects must be expected to induce conviction with those to whom every kind of restraint is dear. Whenever man obtains power over man, he relinquishes that power with an ill grace. He, said some ascetic philosopher, who has no intention to take away your life, is yet not sorry to have it in his hands. The rod, the lash, the chain, the constraining vest, the fretting hand and leg-strap, are only yielded one by one, with natural tears. But on these things another age will look back with surprise; rejoicing in the amended health, the improved appearance, the more decent demeanour of the victims of measures then exploded; and then, and then only, will it be known *how many* of the most wretched class of lunatics, commonly called "dirty patients," were rendered dirty by neglect, and by neglect alone.

The attendants, therefore, from whom so much is required, and upon whom, although of humble rank, the success of the noblest plans will turn, must receive all the consideration to which their harassing situation and their important duties entitle them. As many as can be spared with propriety from their duties, at one time, must be allowed frequent although short absences. Their remuneration must be such as to induce present content, and enable them to make provision for the years in which they will not have sufficient activity left to continue their peculiar labours. Arrangements should be made for their personal comfort. Their nights, except when they take their turn of night-duty, should be undisturbed. They should eat and drink in peace, if possible; at all events, they should dine in common rooms, one for the male and one for the female attendants; half of each dining at a time, after the toil of carving and distributing the dinners of their wards. Whilst their orderly obedience is to be rigidly exacted, they must be treated with kindness, openness, and confidence; made to understand the reason of what is exacted of them; not too sharply rebuked for occasional faults; and generously encouraged when they show a disposition to perform their arduous duties courageously and humanely.

We have said rather more on this subject than we designed to do on the present occasion; but it is time that it received universal attention; and we can assure those who sit apparently secure in the shade of old institutions, that we think they deceive themselves, if they suppose they can discourage the attempts of which we have spoken, or even creditably abstain from speedily making similar attempts themselves. Public attention is awakening, and public indignation may be aroused. Not to Lincoln alone, nor yet to Hanwell, is the great effort even now limited. Across the Atlantic the benign spirit of modern government has manifested itself. In Dr. Woodward's elaborate Report of the State Lunatic Hospital at Worcester, among many passages which we have read with admiration, none has gratified us more than that in which so experienced a physician states, that of 230 patients only *one* was, at the time of his writing, in personal restraint; and that five only were in strong rooms in consequence of violence; whilst, under a system of such leniency, "the furious and violent have become quiet and docile; the filthy and degraded have become cleanly and respectful; and the circumstances in which they are now situated, contrasted with the condition of suffering and wretchedness in which they formerly were, exhibit great improvement and decided benefit." (p. 58.)

So also, in the Bloomingdale asylum, another American institution, the fiscal concerns and moral management of which are superintended by six governors, Dr. Macdonald tells us that under their auspices the treatment of the insane is so mild and parental, that "it is long since there has been such a thing as a strait-jacket in the establishment;" and he adds, that "the utmost extent of freedom, consistent with safety, has been found the best substitute for these means of restraint."

The language of humanity is everywhere the same. All evidence, whether from *restrainers* or *non-restrainers*, bears on the same point, and wears the same colour.

The following case is from the American report:

"No. 3 is a case of homicidal insanity, the subject of which has been in confinement *thirty-four* years. Before he came to the hospital, he had for more than a quarter of a century been confined in a filthy dungeon, without the comforts of life, with neither bed nor covering to keep him warm, and infested with vermin to such a degree, that he could hardly sleep if the means of comfortable repose had been afforded him. He declares that for *seven* winters he did not feel the influence of fire, and that on one occasion a stout and healthy cock lighted upon a tree by the window of his cell and froze to death; this was the 'cold Friday and Saturday' which, in the recollection of all who felt its influence, was proverbially the coldest season of the cold. During these *three* days he declares he did not lie down or sleep, but kept continually walking to keep himself from freezing. He remained in this solitary and filthy cell, the object of the sport and abuse of every idle and mischievous person who took delight in the rage and violence which he could excite, till removed to the hospital. When he entered this institution he was furnished with a neat and cleanly room, a comfortable bed, and everything necessary for his happiness. He had not been shaved for many years, he had not eaten at table or in company, neither had he used a knife and fork during the whole period of this protracted confinement; he soon, however, relearned their use, and became, to a considerable extent, a civil, quiet man. Although the delusions of insanity remain the same, he is now comfortable and happy, he walks abroad at this time unrestrained, takes great care of the poultry, walks about the town and village in company with others, keeps his room in perfect order, makes his bed in the neatest manner, attends chapel every sabbath, and enjoys life as well as the nature of his delusion will permit." (p. 61.)

We might quote several cases of a like purport. The Lincoln report contains many more; but we can only make room for two or three of them:

"Nos. 547, 549, and 551.—These patients were admitted in January, 1836. They had all been confined in a workhouse for a number of years—say between fifteen and twenty. During this period of time they scarcely knew what it was to be at liberty: I have understood that they were chained both day and night to their bedsteads, and kept in a state so filthy that it was heart-sickening to go near them. They were usually restrained with the strait-waistcoat, with collars round their necks; the collars being fastened with chains or straps to the upper portion of their bedsteads, to prevent them (as I have since been informed) from biting their bedclothes: their feet were chained to the bedsteads with iron leg-locks, to which chains were attached. One of the poor creatures, who no doubt had her lower extremities at liberty, was so deformed from the continued confinement, that she was unable to move about, her limbs having become contracted to such a degree that her feet were drawn up until the soles were even with the lower part of her back: when moved from one room to another, it was necessary for an attendant to carry her. These individuals *have never been personally restrained since their admission:* one of them I once placed under seclusion for a few hours only; but beyond that, no coercive means whatever have been employed. Two of these patients have been restored to habits of cleanliness:—one in particular now spends the greater part of her time in knitting, sewing, &c. Of course they have not been so orderly in their conduct as many of their companions; but is this to be wondered at, when for such a number of years they had been treated more like brutes than human beings? The crippled patient died a few months since of consumption.

"1838, April 12.—No. 662, æt. 20.—This patient has been readmitted this afternoon. She was brought in a strait-waistcoat, in a state of the greatest excitement. Five persons could scarcely bring her. She is single, and a member of the Baptist persuasion. This attack came on about a week since; the former about three years ago, from which she recovered, after remaining in this establishment about three or four months. She raves chiefly on religious topics, and is subject to sudden and violent fits of frenzy. During the former attack she attempted self-destruction by jumping into a stone-pit; she occasionally destroys her wearing apparel. Grief and religious excitement are assigned as the immediate exciting causes of the attack. 8 p.m.—She has been very active in her personal exertions, and is unable to control herself.

"April 13.—She has been under watch, and has been restless the whole of the night; she is still very active in her personal exertions, but more tractable than she was yesterday.

"April 14.—She has become quiet and orderly.

"April 15.—She continues calm and well-behaved.

"April 18.—The patient who was brought here on the 12th instant, confined with a strait-waistcoat, and guarded by several attendants, is already so far recovered as to have lost all disposition towards any inordinate action, and has been removed this morning to the moderate patients' gallery. It cannot be doubted, indeed she has herself stated, that *the irritation of personal restraint had occasioned the excitement she at first exhibited.* The strait-waistcoat was instantly taken off on her admission.

"April 20.—She was removed to the convalescent patients' apartment, and is now employed in needlework.

"From April 20 to June 11, inclusive.—Employed in household-work, needlework, &c. &c.

"June 11.—She has been discharged this day by the weekly board of governors. When discharged she applied for the situation of kitchen-maid, and was engaged." (p. 24.)

If Mr. Hill's cases lose any portion of their persuasive effect in consequence of his own zealous and successful efforts (a most unjust but not uncommon consequence), the testimony of those is not wanting who, without being committed to the abolition of restraint, are well known to have endeavoured to improve the management of lunatics. The excellent management of the Nottingham asylum, under its able and humane superintendent Mr. Powell, is so well known, that we cannot but regret to see the somewhat offensive term *Utopian* applied in the Nottingham Report to Mr. Hill's praiseworthy plans; plans which, if they were even to fail, it would be no dishonour to have tried. But it is nevertheless evident, that in the Nottingham asylum, except for protection, restraint is nearly, if not quite unknown. If a patient, to whom the effectual action of a blister is of great consequence, will not refrain from pulling it off, the application of some restriction to the hands for a few hours can be considered no infraction of the non-restraint system. Other cases may be supposed in which the freedom of the hands may temporarily be encroached upon with convenience. That cited in the Nottingham Report is quite satisfactory. A patient had divided several rings of the trachea, and directed every effort to tearing open the wound. In such a case, we agree with Dr. Blake and Mr. Powell, that mechanical restraint was not only justifiable, but necessary; but we should call this *protection*, not restraint. If we were to call this restraint, we should call iron window frames, strong doors, and locks and keys restraint. It is the substitution of habitual corporeal restraint, severe, irritating, long-continued, in the place of practicable superintendence, against which Mr. Hill and all non-restrainers contend; and, as far as we are able to judge from the Nottingham Report, the condemnation of such restraint would be pronounced with no more earnest voice than that of the directors of that well-conducted institution.

We do not suppose, either, that there ever was a time in which the asylum at Hanwell was directed by those who thought restraint, even to the extent to which it might at any time be practised, anything better than a necessary evil. No stronger cases could be adduced against it than those cited in Sir William Ellis's Treatise.

"I know an instance," he says, "where, from continued confinement day and night for years, the limbs had become contracted, the fingers twisted over each other, and the patient totally insensible to the calls of nature. Two stout, ignorant servants, neither of whom could read or write, had been the constant attendants. The maniacal violence and impatience of restraint with which the commencement of the disease was characterized, seemed to have banished from their minds every idea of treating the poor sufferer with decency or respect: and when the first violence of the attack had subsided, no solace was offered to the feelings of wounded pride; but a constant source of irritation remained, in the being obliged to submit to the domination of such associates. An airing was sometimes taken, though the miserable patient, tied hand and foot, was fastened in a blanket to the bottom of the chaise. No wonder that these circumstances should have produced their natural results, and that on an occasional visit from the friends sufficient violence should have been found as apparently to have made such severe confinement necessary. Even this case was not beyond the reach of amelioration. A removal into different society, kind, soothing, and respectful manners, the absence of all restraint, except during the actual continuance of the paroxysm, have rendered the patient cleanly, comparatively happy, and exempt from any exacerbation of the disease for six weeks together. Careful friction of the limbs has restored the use of the muscles, and the patient now enjoys a walk, or a ride, untrammelled. If such be the results where the disease has been of so long continuance, and the mental faculties [have been] apparently destroyed, no case ought to be considered sufficiently desperate to warrant the intrusting the patient at once to the society of the keeper or the nurse, or the neglecting any means which may possibly tend to cure the disease, or to diminish the sufferings."

We cordially concur in these sentiments, and have but to add our expression of concern and surprise, that the institution over which Sir William presided when they were written, cannot be quoted as one in which severe and unnecessary restraints have been unknown. It might have been expected to stand free from the miseries of antiquated institutions.

Never be wearied, we would say in conclusion, to all superintendents of asylums, of devising means to avoid bodily coercion. Never be driven to restrain, by day or by night, one unhappy man or woman, from an apprehension that they *may* do mischief; for such a plan is unjust, cruel, and in the highest degree injudicious; unjust, because it punishes before the fault, and tortures on suspicion; cruel, because it consigns the injured being to dirty habits by rendering him helpless; and, above all, injudicious, because its direct tendency is to impede recovery. Let no one, omnipotent in the lunatic's cell, into which no eye penetrates but that of God, pass these considerations unregarded by; and, leaving his victim in bonds, retire to his own bed self-justified and deceived.

We must here say a few words on the subject of diet. In France, until 1819, lunatics were only allowed bread and water; but their diet is now that of paupers, consisting of soup, meat, and vegetables: wine is allowed in Paris and in the south; cider is given in Normandy, beer in the north. In Germany, on certain days, kept as it were sacred, (des jours solennels) the food is more abundant and delicate than common. It differs much in different establishments in our own country. At Lincoln animal food is allowed daily, but no beer. In some asylums meat is allowed four days in the week, and in some only three, and in very small quantity, seldom more, and sometimes less, than five ounces of meat when cooked. When this is the case, the lunatics have soup three days a week, and meat-pie, or more appropriately pie-crust, with less than two ounces of meat one day in seven. Such, until recently, was the diet-table of Hanwell; but the soup days are now reduced to two, and the quantity of meat is increased. In some asylums beer is freely allowed, in some very sparingly, and at Lincoln not at all. If allowed, it should be of good quality; and without much vigilance the patients will be defrauded of their just quantity, both of beer and bread, especially the imbecile and helpless, who may be half-starved with impunity; not understanding the wrong done them, or unable to represent it. Active patients, who are very useful helpers, are fond of beer; inactive attendants are fond of ease; and a compact is too often made between the parties at the expense of patients who are less alert.

But it is well observed by Mr. Farr, that "the adequacy of the dietary depends to a considerable extent on the amount of nutritive matter in the slops;" and the number of ounces of solid food allowed in a week constitutes the most important particular of comparison. We shall merely observe, that there is the strongest reason for believing that the mortality of those asylums is the greatest where the food is the poorest; and that the recoveries are the most numerous where the diet is the best. The extreme example of what an approach to starvation will do for lunatics is related by Pinel. In the fourth year (of the French revolution), the bread of the patients was reduced from a kilogramme to seven hectogrammes and a half, and by degrees to two: and the mortality which had been but 27 in the whole year, became 29 in two months. Cases of refusal to take food are less numerous than they are commonly supposed to be; and the obstinacy is overcome, in some instances, by mere persuasion; in some by leaving the food with the patient, and saying no more about it. The stomach-pump does not appear to be generally resorted to; but when resorted to its first application is commonly productive of permanent success. All forcible methods of opening the mouth are very objectionable; with the exception of the simple one of closing the nostrils, which plan, aided by the use of a tin vessel with a perforated spout, guarded by a napkin, and introduced between the teeth when the patient separates them to breathe, appears to be the plan pursued when any device is requisite at Lincoln, and in some other asylums. The edges of the spout of these instruments ought, however, to be rounded, to prevent injury to the patient's lips or gums. With every practicable precaution, such devices are never quite devoid of evil. A strong suspicion is entertained by us, that refusal of food is often merely one of the results of the bad treatment to which the patient has been exposed. We believe it is not uncommon for patients to be brought into lunatic asylums, miserable in body and terrified in mind; marked with cords and chains, emaciated, bruised, and even wounded; and refusing, apparently dreading, to take food; and who after experiencing kind treatment for a few days, and once more feeling that they are not utterly deserted in this world, begin to eat and drink again.

The wretched manner of feeding lunatics and idiots, under the old system, when they received each his mess in a bowl or tin, and devoured it like wild beasts, is well contrasted by M. Esquirol with the comfortable manner in which they now take their meals in well-regulated asylums, where well-cooked food is regularly served to them, and they dine together. Their food, however, is not, he thinks, sufficiently varied. Its good quality and comfortable preparation are, doubtless, particularly conducive to the satisfaction and comfort of the patients, but it is by no means easy to induce them all to eat decently; this peculiarity of man, like that of combination of individual power, and of social union, being lost to the human animal when the mind is deranged. Lunatics are not intrusted with knives in every establishment; but this, like many other proofs of confidence, is possible, at least in many cases, where the attendants are sufficiently numerous. M. Esquirol mentions the plan adopted in some of the English asylums of giving them small knives, rounded at the end, the edge being only sharpened in the middle to about the length of three or four inches. Forks with very short prongs are also in common use. The superior safety of these instruments, as weapons of offence, is very doubtful. At Lincoln the food of every patient is cut into small portions in the kitchen. This should always be carefully done for the helpless. The just distribution of the food deserves a more exact attention than it commonly obtains; and weighing and measuring are alone to be depended upon. For patients who lie in bed, the most watchful care is necessary, to prevent their being left without proper food, and, with greater certainty, without their just share of beer.

M. Esquirol makes very few observations on the dresses worn in lunatic establishments. We cannot ourselves approve of the common and uniform garb so often imposed. Every tranquil and convalescent lunatic should at least be dressed carefully, according to his station, and not put into the livery of a mendicant. Like every other source of mental irritation, capable of removal, this deserves consideration. In our variable climate, insane persons should be clothed in woollen, except in the hottest season. Negligence of dress arises in numerous instances from the negligence of the keepers. There are many patients whom no care can induce to keep any covering on their heads.

Repeated allusion is made by M. Esquirol to the importance of allowing space and exercise to the furious lunatic, whom strict seclusion has often exasperated. For this reason, among others, he prefers a large window opposite the door of each patient's room, which may be opened so as to permit his walking out of it without the necessity of opening the door. In case of necessity it is thus also rendered easier to surprise a dangerous lunatic, by feigning an entrance one way and effecting it the other. When a patient has a large window of this kind, he seldom breaks it; although, in other cases, the windows are often destroyed. An observation of this kind occurs in Mr. Tuke's Description of the Retreat, a work in which, we cannot but repeat, most of the enlightened and humane principles of treatment, now generally recognized, will be found pointed to; although many of them have been tardily acted upon in other asylums. We have always made enquiry respecting the occurrence of window-breaking, and it appears to us that the more simple and cheerful the window, the less hostility do the patients exhibit towards it. The confinement of lunatics in galleries or day-rooms, of which the windows are placed so high that they cannot look out, is most objectionable. Very light wire blinds, about eight inches from the window, in the inside, will protect the glass; and even these are only necessary in some wards.

M. Esquirol very properly considers nothing so minute in the management of lunatics as not to merit his attention; he makes several useful observations on the construction of the privies of an asylum, and is decidedly of opinion that they should be separated from the other buildings; the patients being enabled to reach them by corridors, which, with efficient superintendence, is found to be practicable.

In the arrangement of the beds or couches of the patients, the plan of placing them against the wall has inconveniences. M. Esquirol advises that for the violent they should be so detached from the walls as to be easily surrounded. The bed should either be fastened to the floor, or of such heavy materials as not to be moved by one person. Restless patients delight in dragging their beds to the door, or setting them up on end against the window; and the simple precaution of giving them beds too heavy to be lifted is often neglected where straps and strait-waistcoats are liberally applied to restrain these habits. For patients who cannot be kept clean, a bed with a double bottom is recommended: the lower one of wood, coated with lead, sloping towards the foot of the bed, and there perforated, having a reservoir of wood and lead beneath it; the upper bottom of the bed (*à claire-voie*), on which the straw and bed-clothes are placed, should be two inches from the lower. At Stafford, at Gloucester, and probably elsewhere, a strong ticking in a frame is placed some inches above the perforated bed of wood: each bed has two of these, of which one is daily washed and aired. That plan, we should say, is the best which excludes the unpleasant smell so often found in such apartments. In the Gloucester asylum a strong petticoat is often found useful for the *male* patients, the complexity of whose lower garments is found incompatible with cleanliness. Perhaps the kilt and leggings adopted in the Nottingham asylum are preferable to this: but strict watching and attention are better than *any* contrivance.

We should be at a loss to point out any asylum in which the arrangements for the refractory, or rather for the noisiest patients, are yet so complete as to secure quieter patients from annoyance by day and by night: and there is, doubtless, considerable difficulty in effecting such arrangements, although they are especially desirable. The object is to provide for seclusion without subjecting the secluded to any unnecessary privation of light, air, warmth, space, and kind superintendence.

The neglect of proper means of warming lunatic asylums has been found productive of very bad effects, especially on those who are unable to leave their cells; and as the cells are often kept closed for warmth, the vitiated air has sometimes produced scorbutus and other serious maladies. These evils are at present, we hope, unknown in this country, where the apartments are generally all kept at a comfortable temperature. M. Esquirol condemns the arrangements, not unknown in some of our largest establishments, where may be seen assembled round a large iron cage, surrounding the fire-place, a crowd of lunatics, sitting on benches fastened to the floor, and to which some of the unhappy wretches are chained, to the great annoyance of the tranquil and the convalescent. We hope he is right in his conjecture, that these arrangements are now proscribed in England. An open fire, however, is a pleasant object to many poor creatures incapable of occupation; and in some asylums, the ventilation effected by the chimney is far from unimportant. At the best, an iron guard can only be looked upon as a mechanical substitute for other means of care; but not even the watchfulness of Lincoln is trusted to as a substitute for them. The warmth from open fires is less liable to become oppressive than that obtained by other methods.

In every asylum we are inclined to think the majority of the patients will be found to have a feeble pulse, and a small power of preserving the warmth of the extremities. They are prone to all the inconveniences of a low vitality; ulceration where pressure is made, mortification of parts remote from the centre of the circulation, and an inability to resist disease. These circumstances have a very important relation to the regulation of all parts of their diet and regimen, as well as to their medical treatment.

In a review, in our thirteenth number, of several works on insanity, we observed, that in some of the most recent of these an attempt was made to take more than due credit for the institution of labour among lunatics. At the Salpêtrière, says M. Esquirol, the word *work* resounds unceasingly in the ears of the female lunatics, and their ideas are found by this means to be led away from their distractions; their attention is roused; habits of order are established; and when the patient goes out of the institution, restored to reason, chiefly by this plan of labour, she has also often become mistress of a small sum of money. He speaks of trades being followed by the male lunatics in many institutions; and particularly alludes to the asylums at Sarragossa and at Bareuth; nor does he omit to mention the Scotch farmer, whose method was quoted by Pinel. At the York Retreat the principle was long ago recognized, and to a certain extent acted upon. Much credit is due to the late Sir William Ellis for carrying it into fuller effect, although the employment of many patients in sedentary occupations is not favorable to health; and much pecuniary advantage is not to be expected from the labour of lunatics. To the lunatics themselves the labour is most salutary, being powerfully conducive to convalescence and recovery. The rich are to be pitied for the difficulty, in their case, of finding any suitable employment. If this can be done anywhere, it will probably be in the Crichton Institution; and the results cannot fail to be interesting. M. Esquirol advises gymnastic exercises; but what can be done for men who have been accustomed to hunt foxes four days in a week? We really believe that the best plan would be to consider them as grown children, and to establish schools for them in the asylum, on the model of infant schools, so as to give to their unexercised intellects some degree of power, and if possible to instil, even into the minds of those whose leisure hours have been filled up with pursuits little worthy of reasonable beings, some notion of moral responsibility. The sensual, self-indulgent habits of this class of patients, present a formidable obstacle in the way of their perfect recovery. Truly may we add, that no patients are more willingly abandoned, to any kind of treatment, by their nearest relatives and connexions.

To prevent the continuance of a delusion which can only lead to disappointment and mortification, we must repeat, that the work done in a lunatic asylum must not be calculated upon as very profitable in a mere pecuniary sense. Its true profit is of a remedial character. Six shoemakers, who are not sane, may not make more shoes in a week than two shoemakers of sound mind; and ten tailors, in the same predicament, may furnish fewer garments than three rational workmen; but then the whole sixteen are deriving benefit from the stitching, and welting, and hammering, and sewing, and cutting, and goosing, which is worth sixteen times the wages saved by their employment. If it is expected that the labour of the patients in any lunatic asylum will go far to meet the expenses,—an expectation which seems to have been formed by the French government, who have recently sent a deputation to England to enquire into this matter,—the expectation will most assuredly be frustrated. Nor can it be justifiable to apportion labour to lunatics with such an intention. Their malady, as Dr. Charlesworth has on many occasions justly pointed out, incapacitates them for severe and continued exertions. To exact constant day-labour from them may even counteract the remedial influence which is the chief value of employment. What may be done, however, in the way of employment, is shown by the fact that at Hanwell the number of patients, out of 800, who are usually employed, in some way or other every day, is upwards of 400.

Mr. Tulk, the chairman of the visiting magistrates, in a Report distinguished by such liberal and judicious views of asylum management, as show how eminently qualified he is for the direction of so important an institution, has made the following remarks on this branch of the subject, which cannot have too extensive circulation :

" It has been asserted that many of the patients, by the value of their labour alone, could more than compensate for their various expenses. This assertion, calculated as it is to awaken the attention of the rate-payer, has been thrown out without enquiry, and is a complete delusion. It is not possible to make the labour of the patients in the asylum profitable, and to attempt such a thing would be inconsistent with humanity. Your committee must, in answer to speculations of this sort, repeat that a pauper lunatic asylum is not a workhouse; nor could you, without serious consequences, even if you choose to stifle every generous and kind feeling, attempt to get profitable labour out of an insane patient as you might out of a sane and healthy pauper. On this point your committee are satisfied, not only from the results of available labour in the Hanwell Asylum, but from the experience of similar well-conducted establishments. A few instances will be sufficient to show this. In the month of September, 1839, there were ten patients in the asylum employed in the making of shoes, and during that time eighty pounds of leather were delivered out to them. Their labour for that month produced 152 pairs of shoes. The cost of the leather, with cloth and linings, was 6*l.* 13s. 4d. The value of the shoes, at 1s. 8d. per pair, was 12*l.* 13s. 8d. If the cost of the materials be deducted from this, there is left, as the value of their labour, the sum of 6*l.* 0s. 4d., or about 28s. per week, which as nearly as possible pays the cost in wages and board of the shoemaker who superintends them. The same number of sane workmen, had they been employed for the same time, instead of 152 pairs of shoes, would have produced 960 pairs. Still more remarkable is the profit of coir-picking. Of coir-pickers there are, upon an average, 150, whose annual labour produces no more than about the sum of 5*l.* But the value of coir-picking to those patients who can do nothing else, and who, without this simplest of all occupations, must sit or wander about listless all day long, is not to be estimated in money, but must be tried by a far higher standard." (*Fifty-first Hanwell Report*, p. 7.)

The following passages from the Dundee Report afford valuable help towards forming a correct judgment of the real value of the work done in asylums :

" Since the present superintendent and matron undertook the management of the asylum, it has been much distinguished for the variety and extent of the exercises in which the patients have been engaged; and the happy effects of labour on the health and welfare of the patients are now so well known and acknowledged, that many enquiries have been made, and many visits have been paid, by professional and scientific men, to learn the means by which so satisfactory a result had been obtained. The debt was no doubt considerably increased by the erection of workshops and in providing manufacturing utensils; but there is a fair prospect that this debt will be ultimately liquidated and the pecuniary interests of the asylum promoted by the industry of its inmates. The following Table will give a distinct view of the condition of the patients in regard to their ordinary occupations :

Number generally Employed, 1838-39.

| | Males. | Females. | Total. |
|---|---|---|---|
| Weaving linen for sheeting, cotton bagging, &c. | 13 | 6 | 19 |
| Picking oakum . . . . . . . . . | 11 | 0 | 11 |
| Tailoring and mat-making . . . . . | 2 | 0 | 2 |
| Cutting firewood . . . . . . . | 1 | 0 | 1 |
| Mangling clothes . . . . . . . . | 1 | 0 | 1 |
| Pumping water for the use of the establishment | 3 | 0 | 3 |
| Breaking metal for the turnpike road, and gardening, trenching, and laying out ground | 21 | 0 | 21 |
| Domestic purposes . . . . . . . | 1 | 0 | 1 |
| Shoemaking and mending . . . . . | 2 | 0 | 2 |
| Clerks . . . . . . . . . . | 1 | 0 | 1 |
| Dressmaking . . . . . . . . | 0 | 2 | 2 |
| Shoe-binding . . . . . . . . . | 0 | 1 | 1 |
| Spinning . . . . . . . . . | 0 | 11 | 11 |
| Winding for weavers . . . . . . | 0 | 6 | 6 |
| Knitting . . . . . . . . . | 0 | 2 | 2 |
| Shirt-making . . . . . . . . | 0 | 2 | 2 |
| Quilt-making . . . . . . . . . | 0 | 1 | 1 |
| Upholsterers' work . . . . | 0 | 2 | 2 |
| Stay-making . . . . . . . . . | 0 | 1 | 1 |
| Flowering muslins . . . . . . | 0 | 1 | 1 |
| Fringe-making . . . . . . . | 0 | 1 | 1 |
| Repairing bedding and clothes . . . . | 0 | 3 | 3 |
| Worsted works . . . . . . . . | 0 | 1 | 1 |
| Assisting in laundry . . . . . . | 0 | 1 | 1 |
| in scullery . . . . . . . | 0 | 1 | 1 |
| in kitchen . . . . . . . . | 0 | 1 | 1 |
| in bed-rooms and wards . . . . | 0 | 4 | 4 |
| Marking clothes . . . . . . . | 0 | 1 | 1 |
| Total . . . . | 56 | 48 | 104 |

*Ladies and Gentlemen not included in the above.*

*Work done by male lunatics.*

542 Webs of bagging wove.
23 of sheeting, &c.
934 Cwt. oakum picked.
22 Pairs trousers made, in addition to many mended.
13 Waistcoats ditto.
2 Flannel jackets ditto.
14 Coats and jackets ditto.
2 Pairs drawers ditto.
56 Cubic yards metal broke.
55 Pairs leather shoes and boots made, in addition to many mended.

N.B. Garden works cannot be included here, nor cleaning of wards.

*Work done by female lunatics.*

27 Short gowns made.
32 Long ditto ditto.
60 Aprons ditto.
206 Caps ditto.
50 Petticoats ditto.
55 Shifts ditto.
36 Mattresses ditto.
28 Bolster cases ditto.
52 Pillow ditto ditto.
34 Pairs sheets ditto.
18 Ditto stays ditto.
42 Flannel waistcoats ditto.
38 Pairs flannel drawers ditto.
48 Ditto stockings knitted ditto.
81 Men's shirts ditto.
100 Webs sheeting wove.
400 Spindles hemp spun.
260 Handkerchiefs hemmed.
55 Pairs shoes bound.

And winding pirns for 665 webs.

The elegant and other articles prepared by the ladies cannot be inserted here.

"During the erection of the buildings, the patients are busily employed in throwing the foundations, making drains, and in removing earth and rubbish. By their labour several mounds have been erected within the airing grounds, paved on the top, which command a view of the surrounding country, and dispel the monotony and tediousness of a life much secluded from the world.

"The occupations of the higher classes are more varied, and their tastes are gratified in every reasonable wish and healthful exercise. Their amusements and recreations have been particularly specified in former reports: It may be proper, however, now to add, that, for some time, they have been indulged with more frequent excursions into the country; and for this purpose, open and close carriages are provided for them twice a week. There is one fact that cannot be passed over in silence, as it has excited feelings of surprise in the breasts of those who have most experience in the treatment of the insane: A lady of the highest class some time ago expressed a desire that some children were put under her care,—her wish was gratified. A few children from the neighbourhood attend her daily, and she superintends their education, not only with maternal tenderness but with prudence, temper, and judgment; and, amid the many privations to which she is subject, she is conscious that she is not living in vain, but is employing the means within her power of conferring some benefit on our race.

"The Directors have now full experience of the beneficial results that have flowed from the introduction of labour into the asylum, and they have the satisfaction to know that the practice adopted here is closely followed in a number of similar institutions. There is no doubt that these exercises have increased the number of cures, ameliorated the condition of the patient, and shortened the period of the residence in the house; for there are not a few who are now restored to the peaceful bosom of their own families, happy in the consciousness of conferring benefits on others, who without these means would have still been lingering in the airing grounds, sinking under the heavy burden of imaginary evil, or the pitiable victims of illusory hopes. Labour, exercise, and social intercourse, preserve the bodily health of the patients, prevent the mind from brooding over its own disease, and are a most useful associate to the other remedial measures that are usually adopted."

In the latest report of the Retreat at York, the same subject is spoken of with characteristic moderation and good sense:

"The subject of the employment of insane persons in manual labour, with a view to the promotion of bodily and mental sanity, has of late claimed much attention in several asylums, both in Britain and in Germany. The employment of the patients has always been deemed an object of great importance in the Retreat. It has been, from the commencement of the establishment, extensively introduced among the women; and a considerable number of men have also been, from time to time, engaged in the garden, and in other occupations. Particular care has been exercised to endeavour to lead the convalescent patients into some regular employment, adapted to their taste or former habits; and there can be no doubt that these engagements have been highly salutary; but the attempts to introduce a *general system* of employment amongst the men patients have heretofore proved unsuccessful. These experiments have usually been made more with reference to mechanical than agricultural work; but the committee, encouraged by the success of several other establishments, determined to make an effort to induce a number of the men, heretofore wholly unemployed, to engage in gardening and agricultural labour. For this purpose an attendant has been engaged, to take the more immediate charge of the patients employed in labour, and the experiment has now been going on for about three months. The period is too short to determine our permanent success; but we may state that the number of patients who have been induced to work, and the quantity of work done, have both of them exceeded our expectation. Nineteen men patients have been more or less employed during the three months in agricultural work ; the average number so engaged has been about fourteen. They have dug out a grass field of about two acres, and it is now promising a very abundant crop of potatoes and turnips, chiefly the former. In our next report, we purpose entering into a more particular detail of the experiment; we will therefore only add, that the employment has evidently conduced to the comfort of those who have been engaged in it.

"It may be asked: Is it safe to intrust the insane with the requisite instruments for the labour we have described? The answer to this enquiry from eight asylums in Great Britain and Germany, in which the labour system has been introduced, and from which we have obtained particular information, is, that no accident whatever has occurred in connexion with labour ; and they are equally unanimous in stating, that the danger of injury to patients or their attendants, is much greater under the *mere safety system,* than under that which puts spades, rakes, and hoes, into the hands of a large portion of the men patients. Of course discretion is used from day to day in the selection of patients for employment, and care is taken to place them in the charge of suitable attendants." (p. 5).

The Reports of many other institutions now contain allusions to the occupation of lunatics, (those of Aberdeen, Montrose, and the Worcester State Hospital, among others;) but few or none allude to any species of amusement afforded to them, even for the patients of the richer class. It is indeed one of the many disadvantages of persons in the poorer stations, that they have never known any variety of innocent amusements in their lives; and that the very word amusement, when applied to them, awakens associations of unruly behaviour, low gratifications, and sensual irregularity. The strange attempt made long ago at Charenton, and which M. Esquirol relates and stigmatises, to have plays performed by and to lunatics, was both delusive and unsuccessful. We are fearful that lunatic dances will not be found less objectionable, except on rare occasions, and for a short time, and with careful restrictions. Music affords many lunatics a high degree of pleasure. Many who are considered refractory or dangerous, will listen with evident satisfaction to a musical performance. Tears will flow from the eyes of those who show little other sign of sensibility. Yet very few lunatics, among the common people at least, can sing harmoniously; the voice, like the mind, seems "jangled out of tune, and harsh." Playing with a ball, and riding on large and well constructed rocking horses, are the chief amusements of the female patients at Hanwell; and it is found there, as it must be in every pauper asylum, that nothing is more difficult than to furnish a variety of recreation. Various light forms of needlework, feeding poultry, nursing cats and kittens, attending to singing birds, are a kind of substitutes for other amusements among the female patients; but the men are without these resources. Cricket might be practicable; and a bowling-green is found at Nottingham to be a great source of pleasure. We must not presume to mention skittles, which have never enjoyed much magisterial favour; nor cards, which the gambling of the higher ranks has deprived of their inherent harmlessness. Draughts, backgammon, and chess, furnish some with amusement for a few hours in the day. We are now speaking with reference to a class of lunatics, few of whom read with much satisfaction or even facility. For those of higher rank, the resources of amusement are sufficiently numerous. Dr. Macdonald mentions, as adopted in the Bloomingdale asylum, "gymnastics, nine-pins, quoits, the care of domestic animals, draughts, chess, backgammon, dominoes, bagatelle, battledore, the graces, painting, ornamental and plain needlework."

In apportioning the duties of the officers, the principle is not so invariable and simple as M. Esquirol appears to consider it. For instance, he imagines the obvious division of the economic and medical departments to be universally distinct. They assuredly ought to be so, and we presume they are so in nearly every country except our own; where the physician of such establishment is sometimes chiefly valued for his excellence as steward, farm-bailiff, and overseer of works; the office of physician in ordinary being intrusted for the most part to the matron. Of such a state of things, M. Esquirol appears to have no conception.

"In every lunatic house," says he, "the higher functions are divided into two very distinct orders. To the director, steward, or superintending agent, belongs the general administration of the *matériel* of the establishment, the responsibility, maintenance, and execution of the regulations relative to the admission and discharge of patients, and the superintendence of the conduct of the different individuals employed in the establishment (employés). The chiefs of such establishments should have frequent communication with the physician in chief, and have an understanding with him in relation to all the changes and ameliorations that may be required for the interests of the patients confided to their high superintendence. To the physician should be reserved the supreme direction of all that immediately concerns the patients and the medical service."

By this arrangement, the physician would be enabled to attend to his proper duty, *the care and cure of his patients;* which he cannot be if he is steward, and butler, and builder, and farmer, and brewer, and baker, and secretary, and treasurer, and market-gardener. The comprehensive genius of English physicians is alone equal to those multifarious duties. In an institution mentioned by Dr. Crowther, the director was in the habit of visiting the wards once a week, and sometimes only once a fortnight. We may here observe, that Dr. Crowther has very ably drawn the character of a useful physician to such an establishment; and he justly observes, that the number of cures depends upon his unremitting daily labour.

We attach so much weight to every opinion of M. Esquirol, that in addition to the remarks already quoted from him in this and a former number, on the requisite qualifications of a physician to a lunatic house, we cannot but cite his opinion on the same important point in the article on lunatic houses.

"The physician should be, in some sort, the principle of life of a lunatic hospital. By him everything should be put in movement: he directs all the actions, for he is called to be the regulator of all the thoughts. To him, as to a centre of action, should be referred all that interests the inhabitants of the asylum; not only what relates to medicaments, but also what relates to hygiène. The agency of the administration which governs the *matériel* of the establishment, and its superintendence over all the *employés*, ought to be hidden; the director should never appeal against a decision emanating from the physician, and should never interfere between him and the lunatics, or between him and the keepers (*serviteurs*). The physician should be invested with an authority from which nobody should be exempt. . . . . The physician at his visit dictates his prescriptions to a medical and pharmaceutical pupil; the superintendent of the men, and the female superintendent of the women, assist the physician, each in their department; every servant is near the patients, to give an account of them, and to answer questions. The physician ascertains the condition of each lunatic when admitted, and directs his place in the asylum; he orders his removal from one quarter to another; the medical police of the house belongs entirely to him; he prescribes the use of the strait-waistcoat, of restraint or coercion, of baths, of douches; he indicates the kind of amusement, or of occupation suitable to each patient, accords recompenses, &c. &c. With him rests the permission to see the patients; he grants certificates of cure and of discharge, and gives admission to visitors to the interior of the hospital."

It will be observed by every reader, that M. Esquirol considers the treatment of the disease,—of the insanity, as the *first* object in every lunatic asylum. But we must here repeat, what we have before and more than once said, that when we consider the deficient knowledge of many lunatic house-keepers, their total want of acquaintance with medicine, the fulness of their ignorance respecting the human mind, and their mere trading habits, the inevitable result is, a conviction that the restoration of the patient to his senses is the *last* and not the first thing thought of. When an appointment of this kind happens to be vacant, fifty candidates start up for it, with various pretensions, the rarest being that of having even seen a lunatic before. Whole books of testimonials are collected; asked for without a blush, and granted with shameless disregard of the qualifications required: it being too evident that any man who has followed any branch of medicine, or surgery, or pharmacy, is thought fit to undertake the management of the insane.

The sentiments which we have endeavoured to diffuse, supported by the high authority we are enabled to adduce in favour of them, will, we trust, be soon too universally entertained to allow this system, so disgraceful to medical science, and so outrageous to humanity, to exist in any quarter of the kingdom.

The difficulty of procuring proper keepers has been felt by every one who has had the care of lunatics. This office, whilst it offers no compensating advantages, demands a somewhat unusual combination of moral and physical qualities; firmness, forbearance, courage, patience, good sense, humanity, and the utmost promptitude of action, together with sobriety and all the cardinal virtues, are some of the requisites of a keeper who can be intrusted with the details of an enlightened director. Such persons are not easily met with; and servants of lower qualifications are hired at lower rates; and even these are not always sufficiently numerous to pay strict attention to every patient, or to support one another's authority. Their small number in many asylums is evidently compensated for by the habitual restraint of the patients, and by debarring them from proper exercise, and a degree of liberty compatible with efficient watching. In the old Bedlam, says Esquirol, there were five keepers for 120 male lunatics, and two for 120 females. We have found them vary at present, in different asylums, from one to 25 patients to one to 7 patients. In France the keepers are one to 10 patients. M. Esquirol speaks highly of employing convalescents as keepers: they are, generally, he says, compassionate to the infirmities which they have themselves suffered; they aid the physician more efficiently; and their example is encouraging to the other lunatics. The keepers, he adds, should observe a passive and absolute obedience to the orders which they receive in the presence of the lunatics; they should not give an account of such patients as are present; nor should they remain too long in one division of the hospital. To a certain number of them there should be a superintendent of superior education, capable of conversing with the patients, hearing their complaints, consoling them, and encouraging them. In some asylums we have found old military men among the best keepers; keeping up their own authority, and yet obedient to superior orders. With a few such men, having well-selected young men from the country under them, the staff of keepers might generally, we think, be made very effective. Much of the difficulty of procuring efficient persons, male and female, will be overcome where they are liberally paid, and their comfort is in every particular attended to; circumstances indispensable to their cheerful performance of duties demanding sure sacrifices, and even attended with occasional danger. It should ever be remembered, that whatever the skill or diligence of the physician and matron, the keepers and nurses will be paramount in the wards, over the violent and the tranquil, over the feeble and the sickly, over the peevish and the melancholy, for at least sixteen hours out of the twenty-four.

When speaking of the treatment of lunatics in a former number, we had occasion to mention M. Esquirol's humane objections to the rotatory chair, the bath of surprise, and other ingenious inventions for subduing the refractory. He is evidently of the opinion which we have already expressed, that an asylum in which such measures are resorted to, or in which many of the patients are closely shut up, or chained, or confined in the strait-waistcoat, is deficient in moral government and mental discipline. Short seclusions (*la réclusion momentanée*), the camisole (a waistcoat with long sleeves), applied for a short period (*pendant quelques instans*), confinement in an arm-chair constructed for the purpose (*fauteuil de force*), the douche bath long continued, cold affusions, and certain privations, are, he observes, more than sufficient as means of repression, in the hands of a physician who knows how and when to employ them; at favorable times and with moderation: and none of these means should be ordered except by the physician, nor practised except in his presence or that of his head superintendents. M. Leuret cures many patients of their hallucinations by the douche, which he applies until they recant; but we have great doubts of the propriety, or even of the general success of this practice.

M. Pasquier, of Lyons, formerly physician to the hospital of Antiquaille, near that city, into which establishment insane patients, and those affected with cutaneous and venereal affections are admitted, has devoted his attention to the question of the best of the various plans for an asylum devoted to lunatics alone. The hospital of Antiquaille was originally a convent; but its romantic situation, on the declivity of a mountain, however suitable to its first destination, is inconvenient for an hospital for lunatics, offering no facilities for airing-grounds or sufficient divisions, or means of seclusion. In 1821, when M. Pasquier became physician to the hospital, there were one hundred and eighty-two lunatics there, and no kind of classification had been attempted: all that was practicable was the separation of the curable, who constituted five-eighths, from the rest. The violent patients were necessarily shut up until they became calm: the convalescents enjoyed no separation from the rest. The reform of the institution began at this period. Chains and other means of violent repression were abolished. The results of the improved treatment were very favorable; but we can only refer to the second portion of M. Pasquier's pamphlet, in which he lays down a plan for an asylum for 500 lunatics. He considers a building of an octagonal form, with wings radiating from it, as the most convenient; and he objects to the plan of a building with several isolated divisions, as creating difficulties in the way of superintendence, and productive of great expense. We find no writer taking the enlarged view of this point which M. Esquirol does, who conceives that the plan which leads the most directly to the *cure* of the patients may possibly prove to be in the end the cheapest.

The first division which M. Pasquier would recommend would be for the sexes; the second for epileptics; and the others according to the duration and intensity of the symptoms. Consequently there would be a division for the curable, one for the incurable, and one for the convalescents. A division for the younger patients, and a division for those above the class of paupers, he also mentions as desirable. He would further subdivide the epileptic lunatics into the furious and the tranquil. But the difficulties of classification are always greater in practice than on paper; and it would be, we apprehend, not only shocking but hurtful to put all the violent epileptics together. Their distribution among patients, able and not unwilling to aid them in their fits, would seem the kindest way of providing for all epileptics; but even this has some obvious disadvantages. M. Pasquier would subdivide the incurable, so as to separate the paralytic and infirm, those who are dirty in their habits, those who are turbulent, and those who are calm and orderly; and for the latter he would have various workshops. The convalescents would be secluded from all the rest, and placed in the neighbourhood of the physician, apothecary, chaplain, &c., and near the gardens, so as to afford them diversity of amusement. A farm, baths, &c., would form a part of the establishment. The government of the institution, approved of by him, would apparently be nearly that recommended by M. Esquirol. Some excellent remarks are made on the hygienic management of the hospital; but they would not be new to any of our readers. The most important point in the pamphlet, is that which relates to the classification of lunatics, which ought, we are convinced, to comprehend even more subdivisions than are mentioned by M. Pasquier, and many more than exist in any of our public lunatic institutions. As regards the convalescents, especially, we conceive that very careful subdivision should be attended to; and indeed we think the whole subject of convalescent treatment so important, that it might very properly employ the ablest practical pen to treat it properly.

We would suggest the propriety of so arranging the reception-room and wards, that all the new patients, or certainly all the recent cases, and all the tranquil, on their arrival at the asylum, should be exposed as little as possible to sights and sounds capable of aggravating their distress, and making the change insupportable to them. A cheerful room of reception, and direct access to a quiet ward, under the care of a keeper or nurse of good appearance and tried kindness of heart, would make a lunatic house a real *asylum* for many a poor wretch who is now plunged, without remorse, into the most revolting scenes that a refractory ward can furnish.

After these remarks on the latest foreign publications in our hands on this subject, it seems but just to mention that a considerable portion of Sir William Ellis's Treatise on Insanity is devoted to a consideration of the construction of asylums, and the mode of their management. The following we presume to be, in fact, a description of what Hanwell is, or was intended to be:

"The residence of the superintendent and matron, with the various business offices, should be placed in the middle of the centre; and behind these should be the kitchens, sculleries, washhouse, bakehouse, brewhouse, &c. &c., so as to admit of easy access from the centre The wards for the males should occupy one side, those for the females the other side of the building. If the whole of the ground-floor is elevated, which it ought to be, in order that it may be perfectly dry at all seasons, a passage may very easily be made in the basement, from the kitchen to the extreme corners of the central part of the building, along which the provisions, &c. &c., may be conveyed from the various domestic offices, and from these corners to the different wards of both the male and female patients. The gardens, farm-yard, and all other buildings connected with the out-door labour, should be placed at the back of the various offices, from which there should be easy access to them. The airing courts for the wards in the centre of the building, will be on each side of the domestic offices; and, of course, completely separated from each other; those for the side-wings ought to be placed on the east and west sides. If it can be conveniently managed, the entrance to the building should be on the north side, as it is much more cheerful to have the galleries in which the patients walk to front the south; and it is never well for them to be so placed as to be able to see all the persons coming and going to the asylum."

All the observations appended to this description bear upon Hanwell. The healthiness of its site is enforced by the observation, that although few patients are received at Hanwell "until organic disease of the brain has taken place, to such an extent that they are incurable when they are admitted, yet the air is so salubrious, that the deaths, in proportion to the average number of patients in the house, are fewer," &c. &c.

Sir W. Ellis recommends that the building should be of brick or stone, and fire-proof; the roof of iron, and containing cisterns for hot and cold water. He advises a building three stories high. We have seen the many strong reasons advanced against this by M. Esquirol: but, says Sir W. Ellis, "an important saving may be effected by it." We are decidedly of opinion that where this is the general plan of the building, there ought at least to be separate buildings of one story, for the refractory and some other classes of patients; for the sick and for the convalescent; and these ought to communicate with a distinct court or space for exercise. What is proper must not always be sacrificed for what is convenient.

A keeper is recommended as necessary for every twenty or twenty-five patients; and each ward, it is advised, should contain that number only; and for each fifty there should be a dining-room. This number of keepers is decidedly too small for perfect attention to patients in the state called moderate; and quite insufficient for the refractory, for whom the keepers ought to be as one to six or seven. The larger the wards are, the more difficult becomes the proper subdivision and classification of the patients. Large wards, with a keeper's room at one extremity, are, in many respects, inconvenient.

A great variety of minute details are given by Sir. W. Ellis respecting the plan of the house, which are of much interest and importance, but must be read in full to be clearly understood. The regulation of the asylum, as described by him, is more connected with the subject of our present observations. We observe, however, that the establishment of a "walk for spinning string" is strongly recommended, as conducive to the comfort of the patients; and where the galleries, or adjoining wards, cannot be converted to this purpose, a covered way, erected expressly for it, is thought important. For every hundred patients there should be, Sir W. Ellis says, about sixty-six sleeping apartments; and the "sleeping apartments for single patients should not be less than eight feet six inches long, and six feet nine inches wide, and twelve feet high." The remarks on the drains, water-closets, &c., the proper regulation of which is so important wherever a number of individuals are collected together, and so especially important where a love of mischief is superadded to negligence, are very judicious. It is advised that none of the doors be panelled; lunatics breaking through such doors with great facility.

The ventilation and warming of a large lunatic asylum is always a subject of anxious consideration; particularly in the present day, when writers attack a stove with as much severity as if the inventor of a means of keeping mankind comfortable were little better than a criminal; and the troubles of hot air rival the proverbial troubles of hot water. The perfect ventilation of a lunatic asylum is especially desirable. Modern science scarcely appears to have mastered the difficulty of giving a constant supply of pure and dry air, of a moderate temperature, and without exposure to draughts, to buildings of large size, crowded with human beings. Although the difficulty of warming a large building with hot water is very great, Sir William Ellis is inclined to think that this might be still most advantageously done by means of a complete apparatus for each ward. "Pipes heated by steam, and passing under the floor of the galleries, after many experiments, appear the readiest and best mode of heating any very extended building, by one or two apparatuses." "Upon a trial of the two plans at the asylum at Hanwell, it was found that the pipes heated by steam attained the temperature of two hundred degrees of Fahrenheit in an hour and a half; and eight hours elapsed before the same length of pipes, heated by hot water, reached the temperature of one hundred and thirty degrees." If the joints of the pipes are made of iron cement, they are said not to give way, and no wet or discomfort is made in the apartments. Dr. Woodward, of the Worcester State Lunatic Hospital, ascribes the healthy state of that institution in a great measure to the arrangements for warmth and ventilation, both of which objects are effected by hot-air furnaces in the basement. He is of opinion that every other mode of warming is objectionable; and that no other assists ventilation. The object, he observes, is to keep up a constant and regular influx of warm, pure air, in such abundance as to change the whole atmosphere of the apartments frequently. He recommends that the furnaces should be placed in the basement, that the air-chambers should be capacious, and the passages large; and thus a considerable volume of air may be forced into the apartments, heated not many degrees above the temperature at which they are to be kept; and thus the whole air may be frequently changed, and the foul air forced out at the ventilating passages. In all cases the external air, and not the air of a cellar, should be used. The proportion of the outlets for bad air must have a relation to the inlets for the good air. All these are points of importance, and, we suspect, not yet fully understood by practical men.

Whilst care is taken to introduce warmth into large buildings, the admission of fresh air is often almost forgotten. A supply of warm air in winter is desirable, whatever mode of warming the rooms and galleries be adopted; and in all buildings intended for the reception of a great number of human beings, the necessity of an abundant supply of pure air should strictly be kept in mind before the general sickliness of the establishment points to the oversight.

There is, perhaps, no institution in which lunatics have been more systematically employed in various kinds of labour than at Hanwell; and where this is done, Sir William Ellis thinks that it is not necessary that the airing grounds should be very large or numerous. We deem this very questionable; and certainly the cheerful character of the airing grounds is seldom sufficiently attended to; their aspect, when all view is excluded by high walls, being at least calculated to aggravate rather than to cure melancholy. If it could be made consistent with vigilant inspection, the airing grounds should be so disposed that the patients should not always see in one view the whole extent of their walks, and the actual narrowness of the boundary. M. Esquirol was particularly struck with the deficient space allotted in English lunatic asylums for the exercise of the patients in the open air. Certainly, the monotony of the airing courts is often grievous enough; gravel below; and high walls around; and the sky above;—not a tree, not a leaf, not a blade of grass to be seen. We should be glad to find the example of the York Retreat followed, by animals of various kinds being kept in the courts; particularly such as would become familiar with the patients, and consequently a source of innocent pleasure, tending even to awaken the social and benevolent feelings. (*Tuke's Description of the Retreat*, *p.* 96.) The disposition of the moderately extensive grounds at Lincoln deserves to be mentioned with approbation: the mere site of the Lincoln asylum is matchless in this country.

Some of the inmates at Hanwell have learnt to occupy themselves in trades with which they were previously unacquainted: six, for instance, have become shoemakers. There are, also, or were, shops for joiners, painters, glaziers, brush-makers, and coopers. Many of the female patients are employed in fancy and plain-work; and we believe every keeper, nurse, and servant contrives to find a helper, and consequently has much official toil done by deputy. The brew-house, the bake-house, the laundry, the kitchens, large and small, thus become so many convalescent wards.

The Hanwell asylum is placed under the management of fifteen of the Middlesex magistrates, who meet there once a month, and also visit it at uncertain intervals, sometimes inspecting the whole and sometimes a part of it. The effect of these visits is represented as being very useful; and we cannot doubt that they are very advantageous. The Reports just published, although they contain allusions to past untoward circumstances, clearly manifest a uniformity of views and sentiments in the governing and medical bodies. Without this, the distraction of the officers would rival that of the patients. It is of the utmost importance to the maintenance of the effective discipline of an asylum, and of that unity of plan which should pervade every department of it, that the magistrates, or governing committee, should not consider themselves as by their office exempt from conformity to the plan, or suppose that they can, without injury to the institution, scatter throughout its wards expressions at variance with the sentiments of the officer to whom, as a temporary chief at least, they have deputed authority; and who, if his plan is defective, may be admonished; if pernicious, dismissed; but must not, amidst his anxious toil and thought, be teazed and thwarted at every turn. Few days will pass without his being troubled by unexpected accidents, and depressed by unavoidable disappointments: if he is also stung by perpetual irritations, he will either seek security in indolence and indifference, or run much risk of becoming qualified for a place in one of his own wards. Even Pinel laments, in bitter terms, the incessant opposition to which he was subjected, and the harassing manner in which his plans were long obstructed by those who had probably formed no conception of his enlarged and disinterested views. Dr. Crowther, in a work noticed in our Thirteenth Number, expresses himself strongly concerning the difficulties incidental to magisterial management, a point on which we took the liberty to differ from him. We repeat, after long experience of committees of private gentlemen, that we should infinitely prefer them, as a governing body, to any exclusive collection of professional men; expecting from them more varied experience, less jealousy, and fewer conflicting prejudices on practical subjects, as well as a smaller disposition to vexatious interference in petty details. Awkward members are to be found on all committees, we do not doubt; but a preponderance of magistrates disposed to narrow views, or afflicted with a suspicion that all whom they employ are in perpetual combination to deceive them, is surely not to be feared in this country. If, unhappily, there should be a disposition to cavil over every act of the physician or superintendent, and to insult some officer or other every month, the asylum would soon exhibit the results of such disturbing forces.

It is evident that Sir William Ellis, who was steward, and treasurer, and farmer, and much besides, was so overladen with duties, that some part of them, and especially the medical and moral, was of necessity delegated to others. Thus, immediately after breakfast, the house-surgeon, who is the physician's assistant, is described as going round the wards and seeing all the patients: he then "makes a report of any new case of sickness to the physician, whom he subsequently accompanies in his rounds: he also makes up all the medicines, and keeps the medical case-book. In the afternoon this officer again regularly goes round the wards," &c. In short, the physician in chief sees the new cases; but the daily and almost hourly inspections, the constant superintendence, the study of the different cases, and the records of them, seem to have devolved upon the assistant. It can scarcely be otherwise; for the physician, under such a mechanical system, must be receiving or paying money, buying stock for the farm, and mindful of the caprices of the market. Nay, the assistant medical officer must become an assistant to the matron also. It is written in Sir W. Ellis's book that "this officer (the house-surgeon) and the clerk, in each week, inspect the stock of linen, bedding, clothes, &c., in each of the male wards, and, comparing it with the inventory, report any deficiency to the matron."

We are well aware that the moral influence exerted over at least half of the patients and all the nurses by the matron is of very great importance. Supposing the physician to possess every requisite for his situation, he must be nearly powerless over a great part of the establishment without the intelligent and philanthropic co-operation of the matron, on whose goodness of heart and firmness of character it will depend whether for that half of the establishment his wishes are fulfilled or frustrated. Female ingenuity and womanly tenderness can alone devise and apply many means of encouraging and soothing the female patients; reanimating them by confidential employments, and influencing the feminine heart by arguments and suggestions which man's less delicate sensibility can scarcely apprehend; so as to promote convalescence by modes of which he could scarcely ever be perfectly master.

The duties, then, of a matron, without encroaching on those of the physician, are, we acknowledge, scarcely less important. Sir William Ellis, with an implied compliment to the conjugal state of very doubtful desert, conceives that these two functionaries should if possible be married. This of course effects the unity of interest so steadfastly advocated by Sir William; but it may also open a door for confederacy of mismanagement. Yet the matron only can fully carry into effect, as regards the female patients, all the designs of the physician. If gifted with benevolence and understanding, her influence becomes beneficially exerted over at least one half of the asylum; and it will often happen that the zeal and uncalculating philanthropy of a woman will accomplish in a few weeks what man, more cautious, would scarcely achieve in as many months. But we should not wish to see the matron the real physician of the house, and to behold the physician in chief up to his knees in the straw-yard, or plagued to death with the accounts of the butcher, and the baker, and the pig-jobber. That the precise line of duty of all the officers of this establishment required some revision at the time when Sir William Ellis's book was published, is pretty evident from such passages as the following; in which, at the same time, some good and convenient arrangements are pointed out:

"The execution of the different orders made by the committee is intrusted to the resident medical superintendent, a physician, and the matron, who are man and wife. When the peculiar circumstances of these establishments are taken into consideration, it seems a most desirable arrangement that the direction of them should be in the hands of married persons; it gives a home feeling to the parties, and prevents the little petty quarrelling and jealousies which are found continually to exist where single persons preside, and each has a separate interest to attend to. These officers have the entire management, under the control of the committee, of the details of the institution, and give the orders for such things as they have received instructions for from the committee, and for any works of necessity that may arise. The medical and moral treatment of all the patients is under the immediate direction of the resident physician and matron; the resident physician also acts as the treasurer to the institution. The resident physician and matron are assisted by the house-surgeon and his wife; the former of whom, immediately after the patients have breakfasted, goes round the wards on both sides of the house, and carefully examines into the state and general health and comfort of the patients, and makes a report of any new case of sickness to the physician, whom he subsequently accompanies in his rounds: he also makes up the medicines, and keeps the medical case-book. In the afternoon this officer again regularly goes round the wards; in fact, his duty consists in the exercising a constant watchfulness over the servants, particularly over the male keepers, and in the becoming intimately acquainted with the character and circumstances of each individual patient, so as to contrive, with the physician and matron, that not an opportunity may be lost of taking advantage of any favorable turn in the disease. This duty is unceasing; it embraces occasional visits, at uncertain times, to the different male wards, before the servants rise in the morning, to see that the keepers do not permit the patients to get up before they themselves are dressed and ready to attend them, and similar visits after the patients are put to bed at night; to take care that the patients' clothes are taken out of their bedrooms; and that the epileptic patients are so secured as to be unable to turn upon the face, without which precaution they are liable to die from suffocation, in case of a fit coming on. It of course also embraces an attendance, in conjunction with the physician, on any special cases of sickness, as often as may be needed. This officer and the clerk, in each week, inspect the stock of linen, bedding, clothes, &c., in each of the male wards; and, comparing it with the inventory, report any deficiency to the matron." (p. 290.)

The patients' library, for which they are principally indebted to the kindness of Mr. Gurney, is in charge of the female storekeeper. The books consist of biographical works, voyages, travels, and anecdotes; which, *with tracts*, are distributed every Saturday for the use of the ensuing week. The Penny and the Saturday Magazines are also taken. With the help of these, the patients are occupied and kept tolerably quiet on Sunday : a day on which, from the absence, it is said, of their customary occupations, but, more truly, from the absence of their keepers, they are generally more troublesome than usual.

In the old times of lunatic asylums, the keepers used to lock up or chain down their patients on Saturday night, that they might keep holiday from Saturday to Monday. The poor maniac was scrubbed with less ceremony than a horse on the Sabbath-day morning, by an under helper, and fed with less courtesy than a dog; he lay in his straw, on that hallowed day, dirty and distressed and disregarded; whilst his keeper was junketing at Lamb's Conduit-house, the Three Hats, or the Apollo Gardens. The grosser parts of this sketch are now effaced; but, if we mistake not, Sunday is yet the day in all the week most marked by neglect of lunatic-patients. Until very recently one half of the nurses and keepers at Hanwell were absent every Sunday, and some of them from Saturday to Monday.

In making the preceding observations on the constitution of Hanwell, as laid down by Sir W. Ellis, nothing is farther from our intention than to arraign his general good sense or his humanity. Of the latter his work affords many proofs, one of which is alluded to in our former Number, when speaking of the desirableness of making some provision for lunatics when cured; and we have much pleasure in referring to another, in his observations on the bazaar in the Hanwell institution; the various kinds of work called for by which have most beneficially employed several patients who could not be made industrious by any other means. The faults on which we have commented are incidental to the great ignorance of the public, relative to the prospect of cure afforded in mental disorders by careful medical and moral superintendence, and a consequent depreciation of the value of services, for which the regulations usually in force make little provision.

That the duties of a superintendent are far higher than those apparently chiefly contemplated by Sir William Ellis, is well known to many who have visited various institutions of our own country; and, still more, of the continent; whilst the elaborate system pursued in some of the latter, (we may particularly instance those of Siegburg and Winnenden,) are known to require such a devotedness of the time and capacity of the physician, as would be quite impossible under the plan introduced by Sir William Ellis into so large an asylum as that of Hanwell. At present, we believe, few vestiges of that plan remain. There is now a steward, upon whom devolve many of the duties formerly attached to the office of the physician; the superintendence of the buildings, the farm, the gardens, the live and dead stock, and the provisions: and the physician is consequently enabled to attend to his proper duties. That part, however, of the old system which was not open to objection has been carefully retained, and, with the exception of some kinds of labour which were found not to pay the expenses incurred by them, the work at Hanwell has undergone no suspension. It is, however, considered more as a remedial than a profitable application of the patients; and the proper office of the keepers and nurses, which had almost merged into that of mere workpeople, has been carefully restored. The first object of the governors of the asylum is the care and cure of the patients; and to this, labour and everything else is made subordinate. With this limitation, the principle of employing the patients in various kinds of work cannot be too highly spoken of. Sir William and Lady Ellis appear to have acted upon it with an energy and an effect seldom equalled. From twelve to forty patients were constantly occupied on the farm, and as many or more in gardening. Half a dozen of the female patients assisted to milk the cows; and eight were employed, under the direction of one sane person, in brewing and baking for the whole establishment of 660 individuals. From sixteen to twenty patients assisted the laundry-maid to wash. One of the two keepers of each ward was in their time a mechanic; and he daily selected, or had intrusted to him, a portion of the patients, whose employment he superintended: the other keeper attended, after a fashion, to the wards, and employed the patients in picking coir, twine-spinning, and in-door employment. The arrangements in the women's wards were of the same kind.

Our observations have exceeded our proposed limits; but it is difficult to be brief on a subject in which the minutest particular becomes of great practical influence and consideration. The claims of the insane on their happier fellow-creatures are many and sacred. In the ruined bodies and minds of many of them we do but behold the last results of all the moral and political evils which cling to ancient social structures, however various in their plan; adorning them, perhaps, in the eye of the unreflecting spectator, and hiding the rents of a decaying architecture; but ever heaping fresh sources of destruction around the foundations, and assisting time to crumble the whole edifice into dust. The varied forms of misery, of privation and neglect, of abandonment physical and moral, which, in various combinations, make up the great unceasing contention for existence revealed to the physician in all his intercourse with all classes below the richest, concentrate their baleful rays upon the madhouse. Poverty there has done its worst; and man is reduced to a state from which, too often, there is no relief but death. From what causes proceeding it is not our task to investigate, but certain it is that if we go from house to house, except in a small section of society, we do but find disclosed the infinite forms of embarrassment and anxious pain. Madness is the climax. Always let it be impressed upon the minds of those who take the charge of lunatics, that they are called upon to exert their faculties, and to regulate their own actions, for the benefit of the most distressed of mankind, whose wretchedness, deep as it is, may yet be aggravated by unkindness; and that, except them and one "who turneth Him unto the prayer of the poor destitute, and despiseth not their desire," a lunatic has no friend. Relatives cast them off; society banishes, and all fear them. Their very amendment is looked on with suspicion; and for them the eye of affection beams no more. To the guardians of the asylums, whither they are driven for protection or cure, is given the high and singular ministry of securing the comfort and happiness of poor and helpless creatures, forcibly repelled from the more vigorous herd. They may still avert suffering from them: they may surround them with many blessings which they have a capability to enjoy, even in their bereaved intellectual state.

Those who are unacquainted with the history of families over which the plague of insanity has fallen, know but a part of the miseries incidental to human beings. If they could behold the accumulated trials of wives, daughters, and mothers, under such circumstances; the immediate privations, the alarm and agitation, the sacrifices long endured for those who repay such devotion with frantic abuse, with an ingratitude the result of disease, but which scarcely the less wounds and grieves the hearts of those who still love the doomed and falling creature, whose sense and whose character are alike undergoing ruin; they would be convinced that there is no sorrow like their sorrow. Sudden accidents fall upon the working-man, too, in which a fall, a blow, a wound, immediately injures the brain, and incapacitates the honest labourer, yet in the prime of life, from all future profitable work: and who can see and talk to this victim of calamity, in the quiet moments and intervals of his malady, when his anxious thoughts turn with honest faith to his home, to his wife, to his children, without commiserating that ruined humble household; not forgotten, but no longer supported and defended by the unfortunate husband and father, who must linger out his life in an asylum.

It may be thought that madness, like death, knocks alike at the palace gate and at the labourer's hovel; but ever more heavily and darkly does the misery which it flings over devoted households fall on the poor. In the families of the rich, individual eccentricities of temper and manner, and wild and wasteful extravagance, are borne with comparative ease. They are sources of vexation more than of suffering. Abundant comforts and sources of diversion are ever at hand; and however much the erratic relative may be a plague to his connexions for a time, complying doctors are not long wanting, who, called upon by gay impatient wives, or proud and very much shocked relations near of kin, consign the delirious brother or phrenetic husband to an asylum, far away from every acquaintance of his prosperous estate. When he dies, his wealth is divided with a dignified satisfaction; elegant mourning gives assured testimony of a respectable degree of grief, and devout thanksgivings are breathed over superb prayer-books for the comfortable release. Among the poor, the progress of such a case, and its long dragging consequences, are more harassing. Eccentric conduct brings various kinds of punishment on the incipient lunatic himself; blows, and impositions, and imprisonments, and contumely. By degrees his whole family, his hapless children, his affectionate brothers and sisters, or a poor devoted wife, are reduced by successive sacrifices to the lowest condition of poverty. The cottage is disfurnished. Food becomes scanty. With slow-moving charitable rescue, the parish-officers relieve them of their heaviest burden. Yet when, at length, the poor creature, who brought all this suffering upon them, dies in the pauper lunatic asylum, these faithful relatives may be seen, in faded funeral garments, and at an expenditure for the day of a sum, small indeed, but to them considerable, their day's earnings, and sometimes by the omission of their day's food, following to the unregarded grave the remains of the unhappy wretch whose release is truly a mercy; shedding honest tears, for which, as in more exalted stations, there is not the consolation of property inherited, or the consciousness of a graceful sorrow.

In every point of view, then, it is difficult to conceive a task more important, humanly speaking, than that undertaken by the superintendents of lunatic asylums; to whom are committed for repair the spoiled minds of society, with the hope that they can restore the delicate balance, the loss of which has disabled the man for all his duties in society, and made him useless or dangerous; and has cast out the woman from the circle of affections and of decency, pitiable, and distracted, and degraded. Their accomplishments, derived from nature and cultivation, should be proportionate to the undertaking. Their character, whatever it is, will diffuse itself over the household, and exercise a secret influence on every keeper and nurse, and on every patient in whom any trace of sensibility remains. The responsibility hence resulting is enormous. Good sense and good temper, so essential in every social office, are doubly essential here. To which should be added, the most exact order, enlarged views, so surely based as to be unswayed by opposition or difficulty; composure of temper in the midst of agitation, and not to be disturbed by the most violent and undeserved abuse, or by the not unpardonable foibles of agents unprepared by education and habits to fulfil at all times all their intentions. Their duties are not only important but incessant. It is not only that every moment may bring an accident, and every advancing step may be the herald of agitation; but the minds of which they have the charge are never stationary, but advancing or retrograding every hour. To examine the new patients, to watch their progress, to detect the first glimmering light of convalescence, and watch it into day; to remedy the body when its disordered functions manifestly prevent recovery, and to rouse the mind when it lies under a fancied load, and retains a power and wants the will to be exerted : these are a part of their duties, and they comprehend particulars too numerous for any written detail; too constant, too anxious, too serious for any human witness to estimate. They are also often the medium of reunion between the patients and their friends and families; and even when the convalescent is discharged, can scarcely fail to look after their steps for a time with kind solicitude. Nothing can bear the officers safely and well through all these exertions but a sense of duty. Fame will scarcely reward them ; and their department of exertion implies an abandonment of most of the worldly advantages which stimulate ambition beyond the boundaries of the asylum.

But, even without these reflections, the superintendents of a lunatic asylum find their hearts appealed to on every side. To them an hundred helpless hands are held out; and many a faltering, palsied tongue addresses its petitions. Under their management hope revives, even in the cell, and on the bed of straw, and smiles relight the faces of those before forlorn and dead to every joy. By their care the frantic outrage of the maniac is abated, and the unspeakable wretchedness of the melancholic is diminished; by their timely and soothing words the awful dreams of the visionary, who "sees horrid night the child of hell," are oftentimes charmed away. Every act of their benevolence produces its palpable good. Every word, every look of kindness, finds its way to some pained heart, and does its blessed office. The great end, too, of all their exertions,—the restoration of mental power, is infinitely noble. The physician feels that to restore health of body is an elevated art, the value of which those best can appreciate who have ever wanted the blessing. The art of the mental physician is to restore alacrity of attention, readiness of memory, warmth of imagination, accuracy of judgment, and the power to will and to do; the loss of all which is the most grievous part of sickness.

If, then, the abnegation of self in those who take the charge of lunatics is expected to be almost complete, it is that they may be intrusted with the administering of aid to minds more imperfect than their own, and wholly secluded from the cheerful ways of reasonable life. In a world full of common duties, they are separated and devoted to some which may be said, without exaggeration, to be among the highest which a sentient and intellectual being can be privileged to fulfil.

To perform these duties efficiently, they must literally live with lunatics. Constant association with the wild minds that diversify the wards of an asylum can alone give a mastery over them, in every changeful mood. There is nothing to despise in such an occupation. To create the mind has been pronounced a work worthy of the Divinity, and to describe it the highest reach of philosophy: it is no mean task, therefore, to disencumber it of its physical oppressions, to recal its wanderings, to dispel its phantoms, and restore so high a work to unembarrassed exercise.—Perhaps a still more important task yet remains to teach mankind the *causes* of these most fearful visitations; that they may also learn the means of avoiding afflictions difficult to cure, and of which the tendency is to accumulate in every successive generation.

